# Microvascular complications identify a specific coronary atherosclerotic phenotype in patients with type 2 diabetes mellitus

**DOI:** 10.1186/s12933-022-01637-y

**Published:** 2022-10-15

**Authors:** Rocco A. Montone, Dario Pitocco, Filippo Luca Gurgoglione, Riccardo Rinaldi, Marco Giuseppe Del Buono, Massimiliano Camilli, Alessandro Rizzi, Linda Tartaglione, Gaetano Emanuele Rizzo, Mauro Di Leo, Andrea Flex, Michele Russo, Giovanna Liuzzo, Giulia Magnani, Riccardo C. Bonadonna, Diego Ardissino, Filippo Crea, Giampaolo Niccoli

**Affiliations:** 1 Department of Cardiovascular Medicine, Fondazione Policlinico Universitario A. Gemelli IRCCS, L.go A. Gemelli, 1, 00168 Rome, Italy; 2 Department of Internal Medicine, Fondazione Policlinico Universitario A. Gemelli IRCCS, Rome, Italy; 3 Diabetology Unit, Fondazione Policlinico Universitario A. Gemelli IRCCS, Rome, Italy; 4 Department of Cardiovascular and Pulmonary Sciences, Catholic University of the Sacred Heart, Rome, Italy; 5 Department of Medicine and Surgery, University of Parma, Parma, Italy

**Keywords:** Coronary artery disease, Diabetes mellitus, Diabetic microvascular complications, Optical coherence tomography, Prognosis

## Abstract

**Background:**

Patients with type 2 diabetes mellitus (T2DM) are considered as a homogeneous cohort of patients. However, the specific role of diabetic microvascular complications (DMC), in determining the features of coronary plaques is poorly known. We investigated whether the presence of DMC may identify a different phenotype of patients associated to specific clinical, angiographic, optical coherence tomography (OCT) features and different prognosis.

**Methods:**

We prospectively enrolled consecutive T2DM patients with obstructive coronary artery disease (CAD) at their first coronary event. Patients were stratified according to the presence or absence of DMC, including diabetic retinopathy, diabetic neuropathy, and diabetic nephropathy. OCT assessment of the culprit vessel was performed in a subgroup of patients. The incidence of major adverse cardiac events (MACEs) was assessed at follow-up.

**Results:**

We enrolled 320 T2DM patients (mean age 70.3 ± 8.8 years; 234 [73.1%] men, 40% acute coronary syndrome, 60% chronic coronary syndrome). Patients with DMC (172 [53.75%]) presented a different clinical and biochemical profile and, of importance, a higher prevalence of multivessel CAD (109 [63.4%] vs. 68 [45.9%], p = 0.002). At OCT analysis, DMC was associated to a higher prevalence of large calcifications and healed plaques and to a lower prevalence of lipid plaques. Finally, MACEs rate was significantly higher (25 [14.5%] vs. 12 [8.1%], p = 0.007) in DMC patients, mainly driven by a higher rate of planned revascularizations, and DMC predicted the occurrence of MACEs (mean follow-up 33.4 ± 15.6 months).

**Conclusions:**

The presence of DMC identifies a distinct diabetic population with more severe CAD but with a more stable pattern of coronary atherosclerosis.

**Supplementary Information:**

The online version contains supplementary material available at 10.1186/s12933-022-01637-y.

## Background

Type 2 diabetes mellitus (T2DM) is a metabolic disorder representing a major public health problem worldwide [1]. Patients with T2DM are at heightened risk of adverse and cardiovascular (CV) events and, moreover, once CV disease develops, T2DM exacerbates progression and outcomes [1].

Traditionally, the injurious effects of diabetes, through complex molecular pathways involving hyperglycemia, insulin resistance and low-grade inflammation, are separated into diabetic macrovascular (coronary artery disease [CAD], peripheral arterial disease, and stroke) and microvascular complications (diabetic nephropathy, neuropathy, and retinopathy) [2].

Previous studies demonstrated that patients with T2DM and diabetic microvascular complications (DMC) are at higher risk of adverse CV events [3, 4], and such risk persists even after taking into account traditional CV risk factors, suggesting that at least some mechanisms underlying diabetic microvascular disease are also relevant to the atherosclerotic process [4]. Nonetheless, evidence linking micro- and macrovascular complications are lacking and the underlying mechanisms remain speculative. In particular, it is still unclear whether the presence of microvascular involvement may identify a particular subset of patients with different CAD features and natural history.

Over the last years, intracoronary imaging modalities have been developed allowing a morphological characterization of atherosclerotic CAD along with the identification of the pathologic substrate of coronary events [5]. Among these, optical coherence tomography (OCT) is a high resolution imaging modality that provides cross-sectional architecture images of coronary plaque morphology and allows a better understanding of mechanisms of coronary events as well as the plaque characteristics also apprising about the risk of recurrent events [6–8].

Therefore, the aim of this study was to investigate whether in T2DM patients with established CAD, the presence of DMC may identify a different phenotype of patients associated to specific clinical characteristics, angiographic and OCT plaque features as well as different long-term prognosis compared to patients without microvascular involvement.

## Methods

### Study population

We prospectively enrolled consecutive T2DM patients without previous angiographically diagnosed CAD undergoing a clinically indicated coronary angiography at the Department of Cardiovascular Medicine, Fondazione Policlinico Universitario A. Gemelli IRCCS in Rome, Italy, from May 2016 to June 2018. We included both patients with chronic coronary syndromes (CCS) as well as acute coronary syndromes (ACS), diagnosed according to the most recent European Society of Cardiology (ESC) guidelines [9–11] (further details on the study population are reported in Additional file 1). Only patients with obstructive CAD (defined as a stenosis ≥ 50% in the left main coronary artery or any stenosis ≥ 70% or fractional flow reserve [FFR] < 0.80 in any other major epicardial vessel) were enrolled.

Exclusion criteria were a previous CV history (defined as a previously diagnosed ACS or previous evidence of obstructive CAD at coronary angiography, history of coronary revascularization, heart failure, any valvular heart diseases more than mild and any congenital heart disease), severe hepatic or renal dysfunction, malignant disease or acute/chronic inflammatory disease, ages < 18 or > 80 years, and electric or hemodynamic instability at admission. Based on these criteria, 53 patients were excluded from the study. During OCT images analysis, 15 patients were further excluded for poor image quality due to large thrombus amount or residual luminal blood.

### Definitions of diabetes and related complications

T2DM was defined according to the American Diabetes Association (ADA) criteria [12] and the length of the diabetic status and medical treatments at admission and at discharge were recorded. Enrolled patients were stratified, at the time of the index hospitalization, in two groups according to the presence or absence of DMC.

Those without a known history of DMC underwent a comprehensive evaluation at the time of admission to detect them. In particular, these patients underwent: (1) an ophthalmologic examination that included fundoscopy or retinal photography and measurement of visual acuity performed by an ophthalmologist; (2) a neurologic examination investigating the presence of sensory, motor, and autonomic symptoms (3) a 24-h urine collection. DMC was defined according to the ADA criteria and included: (1) retinopathy, defined as any diabetes-related eye disease (macular oedema, severe non proliferative diabetic retinopathy or any proliferative diabetic retinopathy) or use of retinal photocoagulation therapy; (2) neuropathy, including any diabetes-related neurological complication (diabetic peripheral neuropathy, diabetic autonomic neuropathy, cardiac autonomic neuropathy, gastrointestinal neuropathies and genitourinary disturbances) and (3) nephropathy, defined as the presence of microalbuminuria (urinary albumin > 300 mg/g creatinine) and/or an estimated glomerular filtration rate < 60 mL/min/1.73 m^2^ [13]. Moreover, we collected the presence of other diabetic macrovascular complications along with CAD, such as atherosclerotic cerebrovascular disease (including previous vascular ischemic stroke, transient ischemic attack or thrombolysis/thrombectomy); atherosclerotic peripheral vascular disease (femoral artery stenosis > 50% or any vascular surgery or amputation) and carotid arterial disease (defined as carotid artery stenosis > 50% or any previous carotid endarterectomy/stenting) [14–16].

Clinical, laboratory and echocardiographic characteristics of all patients included were collected at admission. The study protocol complied with the Declaration of Helsinki and the study was approved by the institutional review committee. All patients gave written informed consent before coronary angiography.

### OCT image analysis

A subgroup of patients underwent OCT assessment of the culprit vessel after coronary angiography procedure. Culprit vessel was defined as the vessel responsible for the acute clinical presentation in ACS patients, or for myocardial ischemia in CCS patients. OCT images were obtained using a frequency domain OCT system. OCT image analysis was performed using an offline review workstation (Ilumien Optis, St Jude Medical) by two expert investigators who were blinded to patients' data (F.L.G. and R.R.). Analysis was conducted both at the culprit site to characterize the culprit plaque and at the other non-culprit segments of the culprit vessel along the entire OCT pullback.

Plaques of the culprit vessel were analyzed using previously established criteria [17] and OCT analysis was mainly focused on detecting the presence of coronary calcifications, lipid plaques and healed plaques.

Plaques were classified into two categories: (1) fibrous (homogeneous, high backscattering region) or (2) lipid (low-signal region with diffuse border).

Lipid-rich plaque was considered when lipid arc was present with > 90° in any of the cross-sectional images within the lipid plaque [6, 16]. Lipid length and lipid arc were measured on the longitudinal reconstructed view and the cross-sectional image, respectively. For each patient, the cross-sectional image with the largest of lipid arc and the thinnest fibrous cap thickness were used for analysis. Lipid index was defined as the product of mean lipid arc multiplied by lipid length.

Calcifications were identified as an area with low backscattering signal and a sharp border inside a plaque and classified as spotty calcium or large calcifications [8, 16]. Spotty calcium was defined as the presence of lesions with maximal arc < 90° and length < 4 mm [17] whereas large calcifications were defined as lesions with maximal arc ≥ 90° or length ≥ of 4 mm [17]. Healed plaques were defined as plaques presenting with one or more signal-rich layers of different optical density and a clear demarcation from underlying components in at least 3 consecutive frames along the entire plaque [18]. The inter-observer Kappa coefficients were 0.90, 0.89 and 0.92 for lipid plaques, calcifications and healed plaques, respectively. The intra-observer Kappa coefficients were 0.90, 0.91 and 0.93 for lipid plaques, calcifications and healed plaques, respectively. In any case of discordance between the two investigators, a consensus was obtained with the opinion of a third investigator (R.A.M.). Details on OCT procedures and image analysis are reported in Additional file 1.

### Clinical outcome at follow-up

All patients underwent a clinical follow-up by telephonic interview and/or clinical check at 6, 12, 24, 36, 48, and 60 months and, of importance, all patients were discharged from the hospital after the index admission with an optimal medical treatment including aspirin, beta blockers and statins up titrated at the highest tolerated doses.

Major adverse cardiac events (MACEs) were defined as the composite of CV death, non-fatal myocardial infarction (MI) and planned coronary revascularization. CV death was defined as sudden death or death preceded by typical chest pain. Non-fatal MI was defined as typical chest pain at rest associated with ST-segment and/or T-wave abnormalities on the electrocardiogram (ECG) and detection of increased serum troponin I levels. Planned coronary revascularization included any vessel revascularization driven by clinical (i.e., stable pattern of typical chest pain at rest, on exertion, or a combination of both) and/or instrumental evidence of ischemia (i.e., non-invasive stress testing without signs of MI) diagnosed after hospital discharge. Thus, all staged procedures in patients with multivessel diseases were not included in MACEs while deaths occurring during the index hospitalization were included.

### Statistical analysis

Data distribution was assessed according to the Kolgormonov–Smirnov test. Continuous variables were compared using an unpaired Student’s t-test or Mann–Whitney U-test, as appropriate, and data were expressed as mean ± standard deviation (SD) or as median [interquartile range (IQR)]. Categorical data were evaluated using the χ^2^ test or Fisher exact test as appropriate. All tests were two-sided, and a p-value of < 0.05 represented statistical significance. Study population was divided in two groups according to the presence/absence of DMC. Moreover, we performed a sensitivity analysis according to the presence or absence of DMC excluding patients with diabetic neuropathy. Indeed, the presence of diabetic neuropathy may represent a referral bias due to silent myocardial ischemia, thus potentially leading to coronary angiography when atherosclerosis is in a more advanced stage.

Comparisons of plaque characteristics at OCT analysis were performed using a generalized estimating equations (GEE) approach to take into account potential clustering of multiple plaques in a single patient. Multivariate logistic regression analysis for the OCT detection of healed plaques, large calcifications and lipid plaques in the OCT subgroup were performed including all variables with a p-value of < 0.05 at the univariate analysis, diabetes duration (months from T2DM diagnosis) and clinical presentation (ACS vs. CCS). Survival curves of MACEs for patients with or without DMC were produced using the Kaplan–Meier method and were compared by log-rank test. Univariate Cox regression analysis was applied to assess the relation of individual variables with MACEs. Cox regression was then applied to identify variables independently associated with MACEs; to this aim, we included in the multivariate model only variables showing P ≤ 0.05 at univariate analysis, along with age, left ventricle ejection fraction (LVEF), sex, multivessel CAD and diabetes duration (months since T2DM diagnosis). All tests were two-sided, and a p-value of < 0.05 represented statistically significant differences. Finally, given the possible cardioprotective effects of metformin therapy, we performed a sensitivity analysis for MACEs according to the presence or absence of DMC including only patients on metformin (alone or in combination with other oral hypoglycaemic medications) at discharge. Similarly, given the well-known different clinical course of patients presenting with ACS than those presenting with CCS, we also performed a sensitivity analysis for MACEs according to the presence or absence of DMC in patients presenting with ACS and in those presenting with CCS. All analyses were performed using SPSS version 20 (SPSS Inc., Chicago, IL, USA).

## Results

### Baseline characteristics according to presence or absence of DMC

We enrolled a total of 320 consecutive T2DM patients (mean age 70.3 ± 8.8 years; 234 [73.1%] men), 192 (60.0%) presenting with CCS and 128 (40.0%) with ACS. At the time of admission, 172 (53.75%) patients presented with at least one of DMC and, in particular, 108 (33.8%) patients had diabetic retinopathy, 118 (36.9%) diabetic nephropathy and 98 (30.6%) diabetic neuropathy (58 patients with one DMC, 76 patients with two DMC, 38 patients with three DMC).

Compared to patients without DMC, patients with DMC had a longer exposure time to T2DM (median diabetes duration 96.0 [IQR 72.0; 132.0] vs. 84.0 [IQR 60.0; 117.0] months, p = 0.011), worse glycaemic control expressed by higher median glycated haemoglobin (HbA1c) values (48.5 mmol/mol [IQR 44.0; 56.75] vs. 46.0 mmol/mol [IQR 41.25; 51.0], p < 0.001), higher fasting glycaemic level (136 mg/dL [IQR 109; 173] vs. 126.0 mg/dL [IQR 101.25; 149.75], p = 0.019], higher serum creatinine (0.97 mg/dL [IQR 0.81; 1.26] vs. 0.91 mg/dL [IQR 0.79; 1.01], p = 0.006) along with a lower prevalence of dyslipidaemia (88 [51.2%] vs. 95 [64.2%], p = 0.019) with lower low-density lipoprotein (LDL) cholesterol levels (69.0 mg/dL [IQR 57.0; 89.0] vs. 79.0 mg/dL [IQR 65.0; 96.75], p = 0.001) and a lower prevalence of obesity (31 [18.0%] vs. 47 [31.8%] p = 0.004). Furthermore, subjects with DMC had less frequently a familiar history of CAD (39 [22.7%] vs. 48 [32.4%], p = 0.050) and a higher prevalence of peripheral arterial disease (37 [21.5%] vs. 19 [12.8%], p = 0.042).

Moreover, patients with DMC were taking more frequently insulin (89 [51.7%] vs. 34 [23.0%], p < 0.001) and less frequently metformin (90 [52.3%] vs. 105 [70.9%], p = 0.001) compared to those without DMC. In addition, subjects with DMC had a higher prevalence of diastolic dysfunction (133 [77.3%] vs. 95 [64.2%] p = 0.010), in particular of grade II or III (49 [28.5%] vs. 24 [16.2], p = 0.009) with higher mean E/e′ ratio values (8.0 [7.0; 10.0] vs. 7.5 [7.0; 8.0], p < 0.001).

Of importance, patients with DMC had also a higher prevalence of multivessel CAD (109 [63.4%] vs. 68 [45.9%], p = 0.002). In a sensitivity analysis excluding patients with diabetic neuropathy, patients with DMC confirmed a higher prevalence of multivessel CAD compared to patients without DMC (45 [60.8%] vs. 68 [45.9%], p = 0.037).

Clinical, echocardiographic and angiographic features of the overall study population and according to the presence or not of DMC are shown in Table 1. Predictors of DMC are reported in Additional file 1: Table S1.

**Table 1:** Clinical, echocardiographic and angiographic features in the overall population and according to the presence or absence of DMC

Characteristics	Overall population (n = 320)	Patients with DMC (n = 172)	Patients without DMC (n = 148)	p value
Clinical characteristics				
Age [mean ± standard deviation]	70.3 ± 8.8	70.7 ± 9.3	69.8 ± 8.1	0.346
Male sex [n, (%)]	234 (73.1)	122 (70.9)	112 (75.7)	0.340
Hypertension [n, (%)]	274 (85.6)	150 (87.2)	124 (83.8)	0.384
Smoking habit [n, (%)]	143 (44.7)	74 (43.0)	69 (46.6)	0.519
Dyslipidaemia [n, (%)]	183 (57.2)	88 (51.2)	95 (64.2)	**0.019**
Obesity (BMI > 30 kg/m^2^) [n, (%)]	78 (24.4)	31 (18.0)	47 (31.8)	**0.004**
Familiar history of CAD [n, (%)]	87 (27.2)	39 (22.7)	48 (32.4)	**0.050**
CKD (eGFR < 60 ml/min per 1.73 m^2^) [n, (%)]	50 (15.6)	30 (17.4)	20 (13.5)	0.335
Clinical presentation [n, (%)]				0.855
ACS [n, (%)]	128 (40.0)	68 (39.5)	60 (40.5)	
CCS [n, (%)]	192 (60.0)	104 (60.5)	88 (59.5)	
Diabetes duration (months since T2DM diagnosis) [median (IQR)]	96 [72; 120]	96 [72; 132]	84 [60; 117]	**0.011**
Diabetic complications				
Carotid arterial disease [n, (%)]	147 (45.9)	77 (44.8)	70 (47.3)	0.651
Peripheral arterial disease [n, (%)]	56 (17.5)	37 (21.5)	19 (12.8)	**0.042**
Previous stroke/TIA [n, (%)]	27 (8.4)	14 (8.1)	13 (8.8)	0.836
Diabetic retinopathy [n, (%)]	108 (33.8)	108 (62.8)	–	–
Diabetic neuropathy [n, (%)]	98 (30.6)	98 (57.0)	–	–
Diabetic nephropathy [n, (%)]	118 (36.9)	118 (68.6)	–	–
Laboratory data				
Fasting glycaemia (mg/dL) [median (IQR)]	131 [106; 161]	136 [109; 173]	126.0 [101.25; 149.75]	**0.019**
HbA1c (mmol/mol) [median (IQR)]	47 [43; 53]	48.5 [44.0; 56.75]	46.0 [41.25; 51.0]	**< 0.001**
Total cholesterol (mg/dL) [median (IQR)]	150.0 [123.25; 178]	149.50 [118.00; 175.25]	151.50 [125.25; 179.75]	0.411
LDL cholesterol (mg/dL) [median (IQR)]	74.5 [60.0; 92.0]	69.0 [57.0; 89.0]	79.0 [65.0; 96.75]	**0.001**
Hb (g/dL) [median (IQR)]	12.4 [11.7; 13.7]	12.4 [11.4; 13.4]	12.4 [11.7; 13.9]	0.532
WBC (× 10^3^/L) [median (IQR)]	7.7 [7.0; 9.1]	7.7 [6.9; 8.9]	7.8 [7.1; 9.1]	0.343
PLT (× 10^3^/L) [median (IQR)]	234.0 [196.2; 269.7]	233.0 [197.5; 272.0]	238.0 [189.0; 270.0]	0.966
Serum creatinine on admission (mg/dL) [median (IQR)]	0.92 [0.81; 1.13]	0.97 [0.81; 1.26]	0.91 [0.79; 1.01]	**0.006**
Troponin T peak (ng/mL) [median (IQR)]	0.07 [0.01; 1.36]	0.10 [0.01; 1.28]	0.06 [0.01; 2.02]	0.541
Echocardiographic data				
LVEF on admission (%) [median (IQR)]	58 [53; 61]	57 [52; 61]	58 [53; 61]	0.620
LVEF on admission < 50% [n, (%)]	64 (20)	38 (22.1)	26 (17.6)	0.313
Diastolic dysfunction [n, (%)]	228 (71.3)	133 (77.3)	95 (64.2)	**0.010**
Grade II or III diastolic dysfunction [n, (%)]	73 (22.8)	49 (28.5)	24 (16.2)	**0.009**
E/e′ [median (IQR)]	8 [7; 9]	8 [7; 10]	7.5 [7.0; 8.0]	**< 0.001**
Therapy at admission				
Insulin [n, (%)]	123 (38.4)	89 (51.7)	34 (23.0)	**< 0.001**
Metformin [n, (%)]	195 (60.9)	90 (52.3)	105 (70.9)	**0.001**
Sulfonylureas [n, (%)]	59 (18.4)	31 (18.0)	28 (18.9)	0.837
GLP1/DPP4-I [n, (%)]	54 (16.9)	28 (16.3)	26 (17.6)	0.759
SGLT-2-inhibitors [n, (%)]	14 (4.4)	7 (4.1)	7 (4.7)	0.774
Aspirin [n, (%)]	181 (56.6)	97 (56.4)	84 (56.8)	0.948
Beta-blockers [n, (%)]	141 (44.1)	76 (44.2)	65 (43.9)	0.962
CCBs [n, (%)]	65 (20.3)	38 (22.1)	27 (18.2)	0.393
ACE-i/ARBs [n, (%)]	188 (58.8)	106 (61.6)	82 (55.4)	0.260
Statin [n, (%)]	187 (58.4)	102 (59.3)	85 (57.4)	0.735
Diuretics [n, (%)]	53 (16.6)	31 (18.0)	22 (14.9)	0.449
Therapy at dismission				
Insulin [n, (%)]	133 (41.6)	96 (55.8)	37 (25.0)	**< 0.001**
Metformin [n, (%)]	208 (65.0)	98 (57.0)	110 (74.3)	**0.001**
Sulfonylureas [n, (%)]	53 (16.6)	28 (16.3)	25 (16.9)	0.883
GLP1/DPP4-I [n, (%)]	51 (15.9)	26 (15.1)	25 (16.9)	0.665
SGLT-2-inhibitors [n, (%)]	13 (4.1)	5 (2.9)	8 (5.4)	0.259
Aspirin [n, (%)]	313 (97.8)	169 (98.3)	144 (97.3)	0.559
Beta-blockers [n, (%)]	314 (98.1)	169 (98.3)	145 (98.0)	0.852
CCBs [n, (%)]	69 (21.6)	40 (23.3)	29 (19.6)	0.427
ACE-i/ARBs [n, (%)]	290 (90.6)	156 (90.7)	134 (90.5)	0.962
Statin [n, (%)]	309 (96.6)	167 (97.1)	142 (95.9)	0.574
Diuretics [n, (%)]	56 (17.5)	33 (19.2)	23 (15.5)	0.392
Angiographic data				
Multivessel CAD [n, (%)]	177 (55.3)	109 (63.4)	68 (45.9)	**0.002**

Bold values are reported for variables that are statistically significant (p < 0.05)

*DMC* diabetic microvascular complications, *BMI* body mass index, *CAD* coronary artery disease, *CKD* chronic kidney disease, *GFR* glomerular filtration rate, *ACS* acute coronary syndromes, *CCS* chronic coronary syndromes, *T2DM* type 2 diabetes mellitus, *TIA* transient ischemic attack, *IQR* interquartile range, *HbA1c* glycated haemoglobin, *LDL* low-density lipoprotein, *Hb* haemoglobin, *WBC* white blood count, *PLT* platelets, *LVEF* left ventricle ejection fraction, *GLP-1* glucagon-like peptide-1, *DPP4-I* dipeptidyl peptidase-4 inhibitors, *SGLT-2* sodium–glucose co-transpoter-2, *CCBs* calcium-channels blockers, *ACEi* angiotensin converting enzymes inhibitors, *ARBs* angiotensin receptor blockers

### OCT analysis of coronary plaques according to the presence or absence of DMC

A subgroup of 96 (30.0%) patients underwent OCT evaluation of the culprit vessel after coronary angiography. Clinical, echocardiographic and angiographic features in the overall OCT population according to the presence or absence of DMC are summarized in Additional file 1: Table S2.

**Fig. 1:**
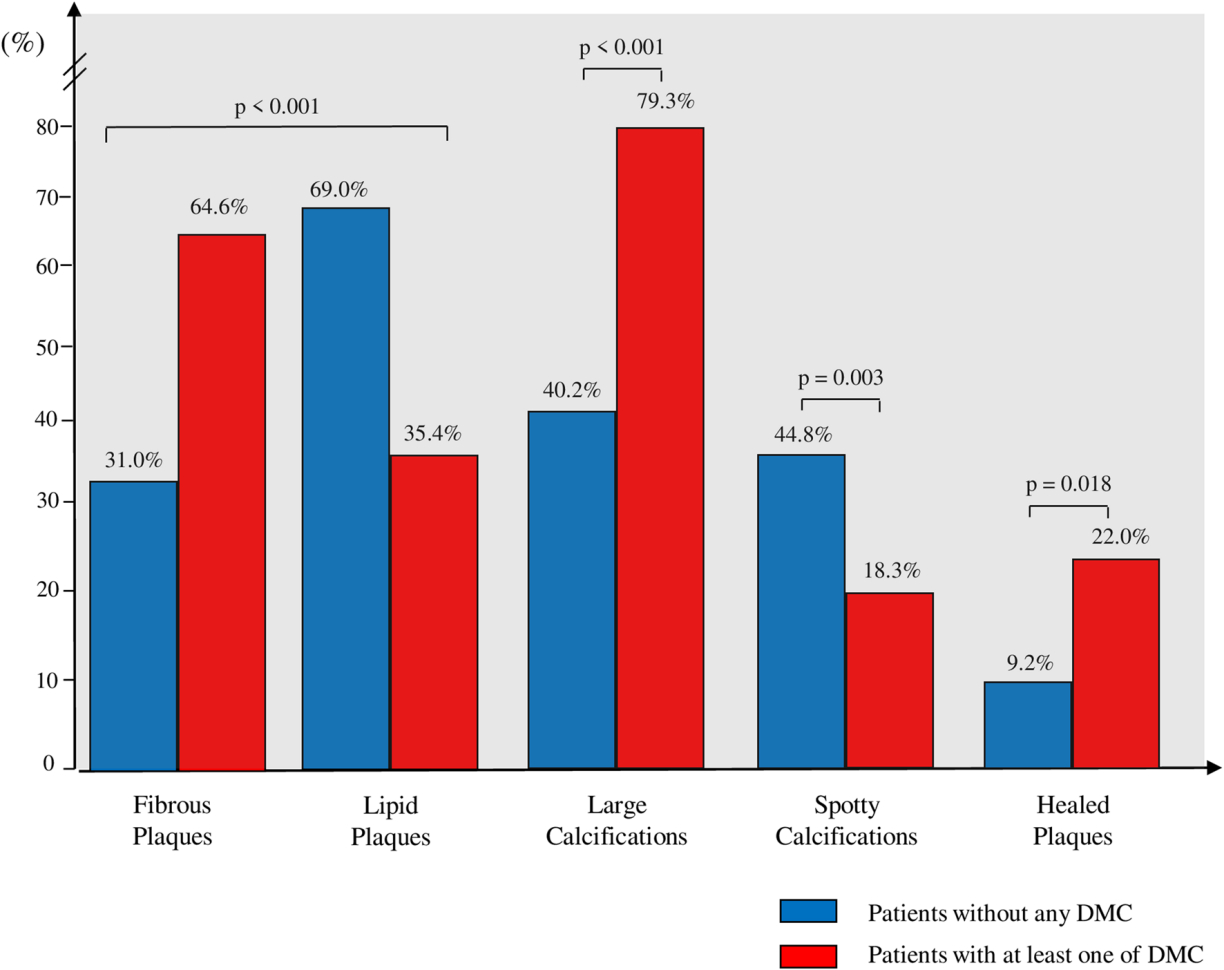
OCT plaque features according to the presence or absence of DMC. *DMC* diabetic microvascular complications

In total, we analysed 169 plaques: 82 (48.5%) plaques of patients with at least one of DMC and 87 (51.5%) plaques of patients without DMC (Table 2). Of interest, coronary plaques of subjects with DMC, compared to those of subjects without DMC, were longer (15.8 ± 7.2 mm vs. 13.5 mm ± 5.9 mm, p = 0.014), more frequently fibrotic (53 [64.6%] vs. 27 [31.0%], p < 0.001) and with large calcifications (65 [79.3%] vs. 35 [40.2%], p < 0.001), along with a higher prevalence of healed plaques (18 [22.0%] vs. 8 [9.2%], p = 0.018]. On the other hand, patients without DMC presented a higher prevalence of spotty calcifications (39 [44.8%] vs 15 [18.3%], p = 0.003) and lipid plaques (60 [69.0%] vs 29 [35.4%], p < 0.001) compared to patients with DMC (Fig. 1).

**Table 2:** OCT characteristics of coronary plaques in the culprit vessel according to the presence or absence of DMC

Characteristics	Plaques of patients with DMC (n = 82)	Plaques of patients without DMC (n = 87)	p value
Clinical characteristics			
Plaque vessel			0.108
LAD [n, (%)]	55 (67.0)	73 (84.0)	
LCx [n, (%)]	18 (22.0)	7 (8.0)	
RCA [n, (%)]	9 (11.0)	7 (8.0)	
Plaque type			**< 0.001**
Fibrous plaque [n, (%)]	53 (64.6)	27 (31.0)	
Lipid plaque [n, (%)]	29 (35.4)	60 (69.0)	
FCT (µm) [median (IQR)]	100.0 (72.5 – 110.0)	100.0 [86.3; 110.0]	0.389
Lipid arc mean (°) [median (IQR)]	168.2 [123.0; 213.5]	185.5 [143.9; 228.3]	0.439
Lipid length (mm) (mean ± standard deviation)	8.7 ± 2.9	9.4 ± 3.7	0.345
Lipid index (mm) (mean ± standard deviation)	1531.2 ± 692.7	1653.1 ± 861.3	0.441
TCFA [n, (%)]	7 (8.5)	13 (14.9)	0.199
Calcifications [n, (%)]	65 (79.3)	35 (40.2)	**< 0.001**
Calcific arc mean (°) (mean ± standard deviation)	165.1 ± 76.6	109.1 ± 107.5	**< 0.001**
Calcific length (mm) (mean ± standard deviation)	10.1 ± 5.0	8.0 ± 4.1	**0.003**
Calcific depth (mm) (mean ± standard deviation)	0.79 ± 0.26	0.52 ± 0.24	**< 0.001**
Spotty calcifications [n, (%)]	15 (18.3)	39 (44.8)	**0.003**
Plaque length (mm) (mean ± standard deviation)	15.8 ± 7.2	13.5 ± 5.9	**0.014**
Healed plaque phenotype [n, (%)]	18 (22.0)	8 (9.2)	**0.018**
Macrophages [n, (%)]	32 (39.0)	36 (41.4)	0.776
Microvessels [n, (%)]	30 (36.6)	29 (33.3)	0.686
Cholesterol crystals [n, (%)]	24 (29.3)	22 (25.3)	0.599
MLA (mm^2^) (mean ± standard deviation)	3.5 ± 2,1	4.0 ± 2.2	0.127
AS (mm^2^) (mean ± standard deviation)	60.9 ± 11.0	58.3 ± 12.4	0.137

Bold values are reported for variables that are statistically significant (p < 0.05)

*DMC* diabetic microvascular complications, *OCT* optical coherence tomography, *LAD* left anterior descending artery, *LCx* left circumflex, *RCA* right coronary artery, *FCT* fibrous cap thickness, *IQR* interquartile range, *TCFA* thin cap fibroatheroma, *MLA* minimal lumen area, *AS* area stenosis

In a sensitivity analysis performed excluding patients with diabetic neuropathy, 128 plaques were included: 43 (33.6%) plaques from patients with DMC and 85 (66.4%) plaques from patients without DMC. Coronary plaques of patients with DMC, compared to those without DMC, were more frequently fibrotic (24 [55.8%] vs. 27 [31.8%], p = 0.029) and less frequently lipid (19 [44.2%] vs. 58 [68.2%], p = 0.029), with a higher prevalence of large calcifications (29 [67.4%] vs. 35 [41.2%], p = 0.031) and healed plaques (10 [23.3%] vs. 7 [8.2%], p = 0.014] (Additional file 1: Table S3).

At multivariate logistic regression analysis, the presence of at least one of DMC (OR 3.838, CI [1.505; 9.786], p = 0.005) and male sex (OR 3.716, [1.091; 12.655], p = 0.036) were positive independent predictors for the presence of large calcifications, while a familiar history of CAD (OR 0.328, [0.114; 0.945], p = 0.039) was a negative independent predictor (Additional file 1: Table S4).

At multivariate logistic regression analysis, only presence of at least one of DMC (OR 3.213, [1.299; 7.949], p = 0.012) was an independent positive predictor of healed plaques at OCT analysis (Additional file 1: Table S5).

Finally, at multivariate logistic regression analysis only the presence of at least one of DMC (OR 0.383, [0.160; 0.918], p = 0.031) was a negative independent predictor for the presence of lipid plaques (Additional file 1: Table S6).

### Clinical outcomes according to the presence or not of DMC

**Fig. 2:**
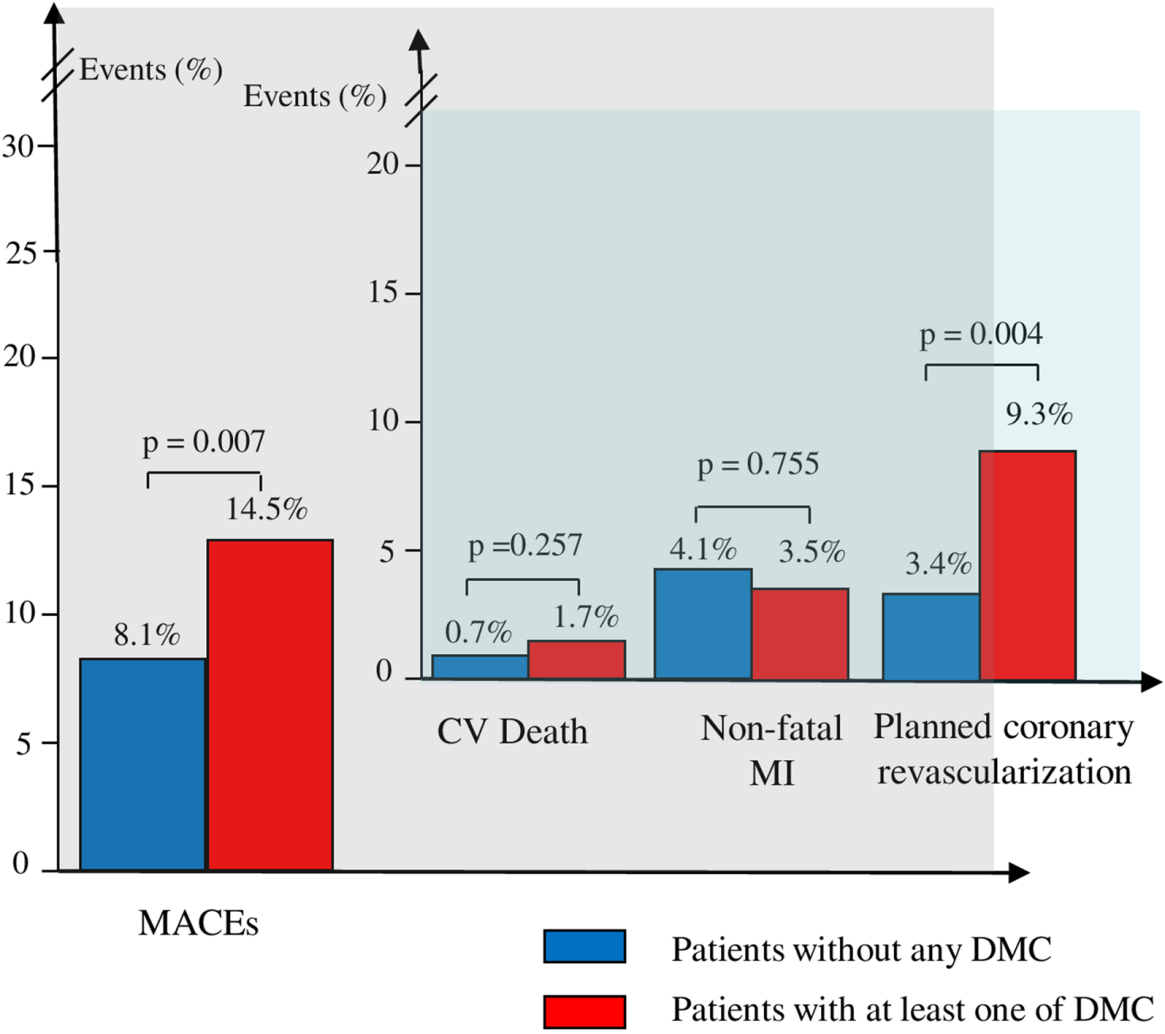
Clinical outcome in the overall population according to the presence or absence of DMC. *DMC* diabetic microvascular complications, *MACEs* major adverse cardiovascular events, *CV* cardiovascular, *MI* myocardial infarction

At a mean follow-up of 33.4 ± 15.6 months, MACEs occurred in 37 (11.6%) patients (Table 3). The incidence of MACEs was significantly higher in patients with DMC compared with those without (25 [14.5%] vs. 12 [8.1%], p = 0.007), mainly driven by a higher rate of planned coronary revascularizations (16 [9.3%] vs. 5 [3.4%], p = 0.004) without differences in the incidence of CV death (3 [1.7%] vs. 1 [0.7%], p = 0.257) and non-fatal MI (6 [3.5%] vs. 6 [4.1%], p = 0.755) (Fig. 2).

**Table 3:** Clinical outcome in the overall population according to the presence or absence of DMC

Characteristics	Overall population (n = 320)	Presence of DMC (n = 172)	Absence of DMC (n = 148)	p value
MACEs [n, (%)]	37 (11.6)	25 (14.5)	12 (8.1)	**0.007**
CV death [n, (%)]	4 (1.2)	3 (1.7)	1 (0.7)	0.257
Non-fatal MI [n, (%)]	12 (3.8)	6 (3.5)	6 (4.1)	0.755
Planned coronary revascularization [n, (%)]	21 (6.6)	16 (9.3)	5 (3.4)	**0.004**
Follow-up time [mean ± standard deviation]	33.4 ± 15.6	32.1 ± 14.9	35.1 ± 16.1	0.088

Bold values are reported for variables that are statistically significant (p < 0.05)

*DMC* diabetic microvascular complications, *MACEs* major adverse cardiovascular events, *CV* cardiovascular, *MI* myocardial infarction

At multivariate Cox regression analysis, both the presence of at least one of DMC (HR 2.891, CI 95% [1.386; 6.030], p = 0.005) and smoking habit (HR 2.049, CI 95% [1.003; 4.187], p = 0.049) were independent predictors of MACEs at follow-up (Table 4).

**Table 4:** Predictors of MACEs in the overall population by univariate and multivariate Cox regression analysis

	Univariate analysis		Multivariable analysis	
	HR (95% CI)	p	HR (95% CI)	p
Presence of ≥ 1 DMC	2.528 (1.261; 5.068)	**0.009**	2.891 (1.386; 6.030)	**0.005**
Smoking habit	2.146 (1.111; 4.144)	**0.023**	2.049 (1.003; 4.187)	**0.049**
Male sex	1.029 (0.498; 2.126)	0.939	0.944 (0.437; 2.038)	0.883
Age	0.969 (0.933; 1.005)	0.093	0.973 (0.936; 1.010)	0.153
LVEF on admission	1.016 (0.975; 1.059)	0.444	1.019 (0.979; 1.061)	0.352
Multivessel CAD	1.015 (0.531; 1.940)	0.964	0.965 (0.473; 1.970)	0.921
Diabetes duration (months since T2DM diagnosis)	0.998 (0.992; 1.004)	0.491	0.997 (0.991; 1.003)	0.319

Bold values are reported for variables that are statistically significant (p < 0.05)

*MACEs* major adverse cardiovascular events, *DMC* diabetic microvascular complications, *LVEF* left ventricle ejection fraction, *CAD* coronary artery disease, *HR* hazard ratio, *CI* confidence interval

All variables in Table 1 and the presence of at least one of DMC have been tested to predict MACEs, although only variables with p-value < 0.050, age, male sex, EF on admission, multivessel CAD and diabetes duration (months since T2DM diagnosis) have been shown in the table. Variables that were significantly related to MACEs, along with age, male sex, EF on admission, multivessel CAD, diabetes duration (months since T2DM diagnosis) have been included in multivariate analysis

**Fig. 3:**
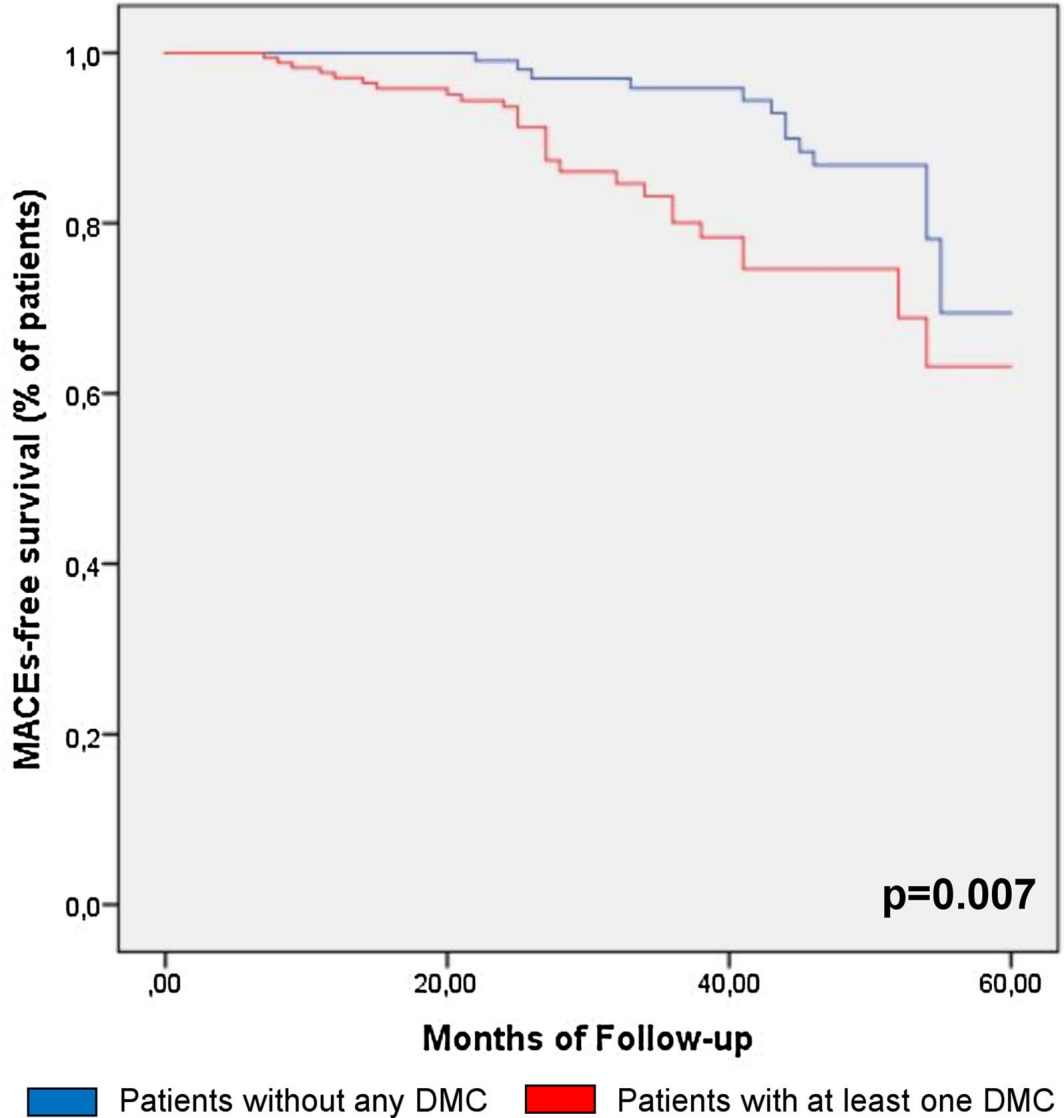
Kaplan–Meier curves for MACEs according to the presence or absence of DMC. *DMC* diabetic microvascular complications

Furthermore, comparison of the Kaplan–Meier curves by log-rank test showed that patients with at least one of DMC had also a lower MACEs-free survival (p = 0.007) compared to those without DMC (Fig. 3).

Moreover, the incidence of MACEs was significantly higher in patients with 3 DMC compared with those without DMC, those with 1 DMC and those with 2 DMC (respectively, 10 [26.3%] vs. 12 [8.1%] vs. 7 [12.1%] vs. 8 [10.5%], p = 0.004) (Additional file 1: Figure S1, Panel A), and the comparison of the Kaplan–Meier curve by log-rank test showed that patients with 3 DMC had also a lower MACEs-free survival (p = 0.004) compared to the other groups (Additional file 1: Fig. S1, Panel B).

In addition, to ascertain the potential effect of metformin on MACEs at follow-up, we also performed a sensitivity analysis including only the 208 patients (98 [57%] with DMC vs. 110 [74%] without DMC) on metformin therapy at the time of discharge. At a mean follow-up time of 33.8 ± 15.8 months, the incidence of MACEs was significantly higher in patients with DMC compared with those without (15 [15.3%] vs. 10 [9.1%], p = 0.025), without statistically significant differences in the incidence of CV death (3 [3.1%] vs. 1 [0.9%], p = 0.133), non-fatal MI (5 [5.1%] vs. 5 [4.5%], p = 0.489) and planned coronary revascularizations (7 [7.1%] vs. 4 [3.6%], p = 0.069), despite the significantly shorter mean follow-up time (31.4 ± 15.6 vs. 35.9 ± 16.1 months, p = 0.043) (Additional file 1: Table S7). At multivariate Cox regression analysis, the presence of at least one of DMC (HR 2.752, CI 95% [1.146; 6.609], p = 0.024) remained an independent predictor of MACEs at follow-up (Additional file 1: Table S8).

Finally, to ascertain the potential effect of clinical presentation on MACEs at follow-up, we also performed a sensitivity analysis including only the 128 patients (68 [53.1%] with DMC and 60 [46.9%] without DMC) presenting with ACS. At a mean follow-up time of 32.2 ± 15.7 months, the incidence of MACEs was significantly higher in patients with DMC compared with those without (11 [16.2%] vs. 5 [8.3%], p = 0.010), without statistically significant differences in the incidence of CV death (2 [2.9%] vs. 1 [1.7%], p = 0.304), non-fatal MI (3 [4.4%] vs. 1 [1.7%], p = 0.171), planned coronary revascularizations (6 [8.8%] vs. 3 [5.0%], p = 0.054) and mean follow-up time (30.3 ± 15.6 vs. 34.4 ± 15.6 months, p = 0.158) (Additional file 1: Table S9). At multivariate Cox regression analysis, the presence of at least one of DMC (HR 3.586, CI 95% [1.146; 11.218], p = 0.028) and smoking habit (HR 5.659, CI 95% [1.419; 22.575], p = 0.014) were independent predictors of MACEs at follow-up (Additional file 1: Table S10). In contrast, in the sensitivity analysis including only the 192 patients (104 [54.2%] with DMC and 88 [45.8%] without DMC) presenting with CCS, at a mean follow-up time of 34.3 ± 15.5 months the occurrence of MACEs had a trend for a higher incidence in patients with DMC compared with those without (14 [13.5%] vs. 7 [8.0%], p = 0.084), with no in CV death (1 [1.0%] vs. 0 [0.0%], p = 0.317) and non-fatal MI (3 [2.9%] vs. 5 [5.7%], p = 0.615) but with significantly higher rate of planned coronary revascularizations (7 [7.1%] vs. 4 [3.6%], p = 0.016) (Additional file 1: Table S11). At univariate Cox regression analysis, no variable was associated with MACEs at follow-up (Additional file 1: Table S12).

## Discussion

In our study, we demonstrate for the first time that CAD occurring in T2DM patients is a heterogeneous process, and the presence of DMC identifies a distinct diabetic population with coronary atherosclerotic features that are different when compared to those of diabetic patients without DMC. In particular, T2DM subjects with DMC at their first coronary event present a more advanced and extended coronary atherosclerosis, with higher prevalence of multivessel CAD at coronary angiography, albeit associated with more “stable” coronary atherosclerosis features at OCT-imaging, as they have a higher prevalence of fibrous plaques and healed plaques, with more frequent large calcifications. In sharp contrast, patients without DMC present a more “vulnerable” plaque phenotype, with a higher prevalence of lipid plaques and more frequent spotty calcifications along with a lower number of healed plaques. Finally, patients with DMC have a higher incidence of MACEs at medium-long term follow-up, mainly due to a higher rate of planned coronary revascularization due to CCS.

Our results are in line with previous studies, showing that subjects with DMC represent a group of diabetic patients with different clinical features compared to subjects without DMC [2, 4, 19, 20]. Indeed, the former group is characterized clinically by a poor glycaemic control, higher insulin requirement, and longer diabetes duration, while the latter is identified by a metabolic syndrome phenotype with insulin-resistance and with a higher prevalence of dyslipidaemia and LDL-cholesterol level, genetic predisposition and obesity [21]. Furthermore, glycaemic variability is narrowly related to the presence of DMC, and an optimal glycaemic and blood pressure control, but not lipid control, has been associated with a lower prevalence of DMC, with no consistent pattern of sex differences reported in the associations of glucose metabolism status and glycaemia with early DMC [22–24]. Moreover, the obstructive sleep apnoea (OSA) is the most common sleep-related breathing disorder that has been associated with the presence of DMC, with the apnoea-hypopnea index that may reflect the association of OSA with DMC, even if we didn’t investigate the presence of OSA in our study [25]. These different biochemical and clinical characteristics may represent the substrate for a different evolution of the atherosclerotic process in patients with T2DM and DMC, with underlying mechanisms mainly related to the injurious effects of hyperglycaemia and insulin therapy (i.e., oxidative stress, endothelial dysfunction, low-grade inflammation, rheological abnormalities and fibrosis activation) [26–28]. Such mechanisms may also explain the higher prevalence of diastolic dysfunction (along with cardiac fibrosis) and multivessel CAD in this subset of patients [27, 29].

Chronic hyperglycaemia plays a central role in the onset of DMC, with specific biochemical pathways linking hyperglycaemia to microvascular changes and fibrosis [30]. Fibrosis, indeed, is the common pathological response to tissue insult such as hyperglycaemia and it is characterized by extracellular matrix (ECM) accumulation due to an increased synthesis of matrix proteins and/or an inhibition of ECM degradation [31]. A longer exposure to chronic hyperglycaemia may trigger low-grade vascular inflammation and oxidative stress through production of free reactive oxygen species (ROS), interaction of advanced glycation end products (AGEs) with their receptors (RAGEs) [32], lipotoxicity, enhanced activation of protein kinase C isoforms and endothelial dysfunction [33]. These biochemical pathways can alter ECM turn-over through the accumulation of multiple growth factors (i.e., transforming growth factor-β [TGF- β], connective tissue growth factor [CTGF], insulin-like growth factor I [IGF-I], epidermal growth factor [EGF] and platelet derived growth factor [PDGF]) along with an imbalance between metalloproteinases (MMPs) and their respective tissue inhibitors (tissue inhibitors of metalloproteinases [TIMPs]) that synergistically favours tissue fibrosis. Furthermore, along with hyperglycaemia, also insulin may have profibrotic effects and, together with the presence of microvascular inflammation and activation of cellular pathways (i.e., transforming growth factor-β production and nuclear factor-κB activation), may promote fibrosis and calcification in different organs as well as in coronary arteries [31–34].

Given these considerations, the presence of DMC may subtend different biochemical and functional mechanisms that could explain the observed clinical and angiographic differences. In fact, these patients showed a more diffuse and advanced CAD at the time of the first CV event, as indicated by the higher percentage of patients with multivessel disease, at first glance suggesting a more aggressive atherosclerotic process and partially explaining the higher associated risk of MACEs at follow-up [2, 3]. On the other hand, we can argue that in patients with DMC the presence of a more advanced and extended CAD at the first CV event, coupled with a higher rate of MACEs at follow-up mainly driven by planned coronary revascularization, may suggest the presence of a coronary atherosclerotic process with more stable features associated with a delayed clinical presentation compared to patients without DMC. Accordingly, our OCT data demonstrated that patients with CAD and T2DM with DMC had a distinct coronary atherosclerotic phenotype.

In particular, these patients had a higher prevalence of healed plaques, with the presence of at least one of DMC being an independent predictor of such particular phenotype. Plaque healing is considered the result of sub-clinical plaque destabilization with non-occlusive thrombosis and it seems to protect from the occurrence of ACS leading instead to a CCS due to progressive plaque growth and lumen narrowing [35]; conversely, impaired healing is associated with ACS and its recurrence [36].

In addition, the presence of microvascular involvement was an independent predictor of large coronary calcifications. The mechanisms of diffuse coronary calcifications in these patients might be related to the above mentioned consequences of chronic hyperglycaemia (in particular low-grade vascular inflammation and oxidative stress) that promote the differentiation of macrophages into a pro-healing phenotype and the transformation of vascular smooth muscle cells into osteoblast-like phenotype [37]. Previous studies revealed that coronary calcifications could have a potentially plaque-stabilizing effect, whereas soft, predominantly lipid-rich lesions with spotty calcifications are hallmarks of vulnerable plaques [38]. Furthermore, diffuse calcifications were found to be more prevalent in healed plaque where they could provide mechanical support for the healing process [33]. Therefore, calcium formation appears to be an active process involved in plaque stabilization and healing [35] that could be somehow more frequent in patients with diabetic microvascular involvement.

Finally, the hypothesis of a “wound healing” plaque polarization with different mechanisms underlying atherosclerotic plaque formation and progression in the specific population is further suggested by the fact that the presence of DMC was found to be an independent negative predictor of lipid plaque phenotype. The presence of a large lipid core is considered the main feature of rupture-prone plaques leading to ACS and its formation and progression is mainly related to traditional CV risk factors, in particular LDL cholesterol levels [39–41].

Taken together, our findings suggest that in patients with T2DM and CAD, the presence of DMC leads to a more diffuse atherosclerotic pattern characterized, however, by plaques less prone to cause acute symptomatic events along with a trend towards healing. Furthermore, this may also explain why patients with DMC, despite the higher prevalence of multivessel CAD at the time of diagnosis, experience their first CV event later in the disease course and have a higher risk of MACEs driven only by target lesion revascularization with similar rates of death from CV causes and non-fatal MI.

### Study limitations

Our study has several limitations. First, it is a single-centre investigation with a relatively small sample size. Second, the decision to perform OCT imaging was at the operator’s discretion and not performed for several reasons (i.e., technical feasibility such as for subocclusive stenosis or extreme tortuosity, need for further administration of contrast medium) and therefore potential selection bias cannot be excluded. Larger studies are warranted to further characterize these features and their potential clinical implications, and these data should therefore be interpreted with caution and considered as hypothesis-generating. Third, in the group of patients without DMC, the higher prevalence of metformin therapy at discharge might contribute to slow atherosclerotic plaque progression [42], even if its cardioprotective role is controversial [43]. However, our sensitivity analysis including only patients taking metformin at discharge showed that DMC remained associated to a higher risk of MACEs at follow-up. Fourth, only baseline predictors were included in the prospective analyses. Moreover, diabetes duration was defined as months since T2DM diagnosis, and we cannot exclude that, in some cases, it may be underestimated due to a late diagnosis. Finally, the diagnosis of DMC (i.e., diabetic retinopathy, diabetic neuropathy, and diabetic nephropathy) was performed according to current guidelines and after a careful exclusion of other potential differential diagnosis (e.g., urinary tract infections, IgA nephropathy and vasculitis to exclude other causes of microalbuminuria or chronic kidney disease; vitamin B12 deficiency and chronic inflammatory demyelinating polyneuropathy to exclude other causes of neuropathy). However, other possible, even though very rare, aetiologies could not be excluded.

## Conclusion

In conclusion, the hypothesis of a different pattern of atherosclerosis in patients with CAD and T2DM with DMC is confirmed by the demonstration that patients with microvascular burden present not only different clinical characteristics but also different plaque features and different atherosclerotic phenotype. Such differences may be due to the prolonged effects of chronic hyperglycaemia on microvascular environment that probably leads to a more stable pattern of atherosclerosis in this subset of patients, as demonstrated by the presence of diffuse calcifications and healed plaque phenotype.

## Supplementary Information

**Additional file 1: Table S1.** Predictors of DMC in the overall population by univariate and multivariate logistic regression analysis. **Table S2.** Clinical, echocardiographic and angiographic features in the overall OCT population according to the presence or absence of DMC. **Table S3.** Sensitivity analysis of OCT characteristics of coronary plaques in the culprit vessel according to the presence or absence of diabetic microvascular complications excluding patients with diabetic neuropathy. **Table S4.** Predictors of large calcifications in the OCT sub-group by univariate and multivariate logistic regression analysis. **Table S5.** Predictors of healed plaque phenotype in the OCT sub-group by univariate and multivariate logistic regression analysis. **Table S6.** Predictors of lipid plaque phenotype in the OCT sub-group by univariate and multivariate logistic regression analysis. **Table S7.** Sensitivity analysis of clinical outcome according to the presence or absence of diabetic microvascular complications including only patients on therapy with metformin at the time of discharge. **Table S8.** Sensitivity analysis of predictors of MACEs including only patients on therapy with metformin at the time of discharge by univariate and multivariate Cox regression analysis. **Table S9.** Sensitivity analysis of clinical outcome according to the presence or absence of diabetic microvascular complications including only patients with ACS as clinical presentation. **Table S10.** Sensitivity analysis of predictors of MACEs in the ACS population by univariate and multivariate Cox regression analysis. **Table S11.** Sensitivity analysis of clinical outcome according to the presence or absence of diabetic microvascular complications including only patients with CCS as clinical presentation. **Table S12.** Sensitivity analysis of predictors of MACEs in the CCS population by univariate and multivariate Cox regression analysis. **Figure S1.** **A** Clinical outcomes in the overall population stratified according to the number of DMC. **B** Kaplan–Meier curve for MACEs according to the number of DMC. Abbreviations: MACEs; Major Adverse Cardiovascular Events; DMC: Diabetic Microvascular Complications.

## Data Availability

All data and methods supporting the findings of this study are available from the corresponding author upon reasonable request.

## Abbreviations

ACS: Acute coronary syndromes
ADA: American Diabetes Association
AGEs: Advanced glycation end products
CAD: Coronary artery disease
CCS: Chronic coronary syndromes
CV: Cardiovascular
CTGF: Connective tissue growth factor
DMC: Diabetic microvascular complications
ECG: Electrocardiogram
ECM: Extracellular matrix
EGF: Epidermal growth factor
ESC: European Society of Cardiology
FFR: Fractional flow reserve
GEE: Generalized estimating equations
HbA1c: Glycated haemoglobin
IGF-I: Insulin-like growth factor I
IQR: Interquartile range
LDL: Low-density lipoprotein
LVEF: Left ventricle ejection fraction
MACEs: Major adverse cardiac events
MI: Myocardial infarction
MMPs: Metalloproteinases
OCT: Optical coherence tomography
PDGF: Platelet derived growth factor
RAGEs: Receptors for advanced glycation end products
ROS: Reactive oxygen species
SD: Standard deviation
T2DM: Type 2 diabetes mellitus
TGF- β: Transforming growth factor-β
TIMPs: Tissue inhibitors of metalloproteinases
