# Supplementary material for: Microvascular complications identify a specific coronary atherosclerotic phenotype in patients with type 2 diabetes mellitus
Source: Cardiovasc Diabetol. 2022 Oct 15;21:211. doi: 10.1186/s12933-022-01637-y (PMC9571474; doi:10.1186/s12933-022-01637-y)
Supplement: Supplementary file 1 — Additional file 1: Table S1. Predictors of DMC in the overall population by univariate and multivariate logistic regression analysis. Table S2. Clinical, echocardiographic and angiographic features in the overall OCT population according to the presence or absence of DMC. Table S3. Sensitivity analysis of OCT characteristics of coronary plaques in the culprit vessel according to the presence or absence of diabetic microvascular complications excluding patients with diabetic neuropathy. Table S4. Predictors of large calcifications in the OCT sub-group by univariate and multivariate logistic regression analysis. Table S5. Predictors of healed plaque phenotype in the OCT sub-group by univariate and multivariate logistic regression analysis. Table S6. Predictors of lipid plaque phenotype in the OCT sub-group by univariate and multivariate logistic regression analysis. Table S7. Sensitivity analysis of clinical outcome according to the presence or absence of diabetic microvascular complications including only patients on therapy with metformin at the time of discharge. Table S8. Sensitivity analysis of predictors of MACEs including only patients on therapy with metformin at the time of discharge by univariate and multivariate Cox regression analysis. Table S9. Sensitivity analysis of clinical outcome according to the presence or absence of diabetic microvascular complications including only patients with ACS as clinical presentation. Table S10. Sensitivity analysis of predictors of MACEs in the ACS population by univariate and multivariate Cox regression analysis. Table S11. Sensitivity analysis of clinical outcome according to the presence or absence of diabetic microvascular complications including only patients with CCS as clinical presentation. Table S12. Sensitivity analysis of predictors of MACEs in the CCS population by univariate and multivariate Cox regression analysis. Figure S1. A Clinical outcomes in the overall population stratified according to the number o [file 12933_2022_1637_MOESM1_ESM.docx]

**Additional file 1**

**Microvascular Complications Identify a Specific Coronary Atherosclerotic Phenotype in patients with Type 2 Diabetes Mellitus**

Rocco A. Montone, MD, PhD^1^*, Dario Pitocco, MD, PhD^2,3^*, Filippo Luca Gurgoglione, MD^4,5^, Riccardo Rinaldi, MD^4^, Marco Giuseppe Del Buono, MD^4^, Massimiliano Camilli, MD^4^, Alessandro Rizzi, MD^3^, Linda Tartaglione, MD^3^, Gaetano Emanuele Rizzo, MD^3^, Mauro Di Leo, MD^3^, Andrea Flex, MD, PhD^2^, Michele Russo, MD^4^, Giovanna Liuzzo, MD, PhD^1,4^, Giulia Magnani, MD, PhD^5^, Riccardo C. Bonadonna, MD, PhD^5^, Diego Ardissino, MD^5^, Filippo Crea, MD^1,4^, Giampaolo Niccoli, MD, PhD^5^

** These authors equally contribute as first authors to this manuscript*

*^1^ Department of Cardiovascular Medicine, Fondazione Policlinico Universitario A. Gemelli IRCCS, Rome, Italy*

*^2^ Department of Internal Medicine, Fondazione Policlinico Universitario A. Gemelli IRCCS, Roma, Italy*

*^3^ Diabetology Unit, Fondazione Policlinico Universitario A. Gemelli IRCCS, Roma, Italy*

*^4^ Department of Cardiovascular and Pulmonary Sciences, Catholic University of the Sacred Heart*

*Rome, Italy*

*^5^ Department of Medicine and Surgery, University of Parma, Parma, Italy*

**Legend**

- **Additional Methods**
- **Additional References**
- **Additional Tables**
- **Additional Figure**
- **Additional Figure Legend**

***Additional Methods***

*Study population, invasive treatment and clinical data collection*

Patients with chronic coronary syndrome (CCS) were defined as those admitted with a stable pattern of typical chest pain at rest, on exertion, or a combination of both, and evidence of ischemia at non-invasive stress testing without signs of myocardial infarction (MI) (1).

MI was defined as the detection of raise and fall of serum high-sensitivity (hs) troponin I levels with at least one value exceeding the 99th percentile of a normal reference population and at least one of the following: symptoms of myocardial ischemia and/or new ischemic electrocardiogram (ECG) changes (namely ST-segment, T wave abnormalities and/or pathological Q waves) and/or imaging evidence of loss of viable myocardium or new regional wall motion abnormality in a pattern consistent with an ischemic etiology (2).

Patients with acute coronary syndromes (ACS) included ST-segment elevation MI (STEMI) and non-ST-segment elevation ACS (NSTE-ACS).

Patients with STEMI were defined as those with acute chest pain, new persistent ST-segment elevation (more than 20 minutes), hs troponin I rise and fall, and/or new regional wall motion abnormalities (3).

Patients with NSTE-ACS were defined as those with at least two episodes of angina at rest or one episode lasting more than 20 minutes during the preceding 48 hours and normal levels (unstable angina) or raise and fall (non-ST-segment elevation MI [NSTEMI]) of hs troponin I levels (4).

All patients with CCS received aspirin (250 mg IV loading dose, followed by aspirin 75 to 100 mg daily) on admission to the Emergency Department and were treated with a loading dose of clopidogrel (600 mg) before percutaneous coronary intervention (PCI).

All patients with STEMI were treated with aspirin (250 mg intravenous [IV] loading dose, followed by aspirin 75 to 100 mg daily) and a loading dose of a second antiplatelet drug [ticagrelor (180 mg) or prasugrel (60 mg) or clopidogrel (600 mg), according to physician’s choice] on admission to the Emergency Department.

All patients with NSTE-ACS received aspirin (250 mg IV loading dose, followed by aspirin 75 to 100 mg daily) and were treated with a loading dose of a second antiplatelet drug [ticagrelor (180 mg) or prasugrel (60 mg) or clopidogrel (600 mg), according to physician’s choice] and fondaparinux or enoxaparin on admission to the Emergency Department.

PCI were performed through a radial or femoral access according to operator’s preference, using a 6 French catheter. A weight adjusted IV bolus of 100 IU/kg of unfractionated heparin was administered at the time of PCI, with repeat boluses to achieve an activated clotting time of 250 to 300 seconds. The use of glycoprotein IIb/IIIa inhibitors or manual thrombus aspiration was left to operator’s decision.

Finally, cardiovascular risk factors were carefully examined in all patients, including family history of early coronary artery disease (first-degree relative with a history of myocardial infarction <60 years), diabetes mellitus, hypercholesterolemia (total cholesterol >200 mg/dL or treated hypercholesterolemia), smoking (current), and hypertension (systolic blood pressure >140 mmHg and/or diastolic blood pressure >90 mmHg or treated hypertension). Body mass index was also obtained. Routine laboratory data, including full blood count, serum creatinine, glomerular filtration rate, glycaemia, lipid profile, glycated haemoglobin (HbA1c) and admission creatin-kinase and hs-troponin I were also collected. Medications taken on admission and dismission were recorded.

*Optical Coherence Tomography (OCT) procedure*

Coronary angiography was performed via the transradial or transfemoral approach with the use of a 6F sheath, followed by OCT imaging of the culprit vessel. Unfractionated heparin (initial weight-adjusted intravenous bolus of 60 IU/Kg, with repeat boluses to achieve an activated clotting time of 250 to 300 seconds) was administered in all patients. The use of intracoronary or intravenous glycoprotein IIb/IIIa inhibitors was left to the operators’ discretion. A 0.014-inch guidewire was placed distally in the target vessel and an intracoronary injection of 200 µg of nitroglycerine was performed. In the overall cohort of patients undergoing coronary angiography for ACS the decision to perform OCT was left to operator’s decision. Frequency domain OCT (FD-OCT) images were acquired by a commercially available system (C7 System, LightLab Imaging Inc/ St Jude Medical, Westford, MA) connected to an OCT catheter (C7 Dragonfly; LightLab Imaging Inc/ St Jude Medical, Westford, MA), which was advanced to the culprit lesion. The FD-OCT run was performed using the integrated automated pullback device at 20 mm/s. Coronary angiography was performed within 90 minutes from hospital admission in patients presenting with STEMI, and within 48 hours in patients presenting with NSTEMI. High-risk patients presenting with hemodynamic instability underwent coronary angiography within 120 minutes. OCT was performed after coronary angiography. In particular, in case of STEMI patients, OCT was performed after vessel reopening with wire passage or thrombus aspiration followed by gentle predilatation if necessary. In case of NSTEMI patients OCT was performed either directly or after gentle predilatation where OCT probe could cause a luminal obstruction. During image acquisition, coronary blood flow was replaced by continuous flushing of contrast media directly from the guiding catheter at a rate of 4 ml/s with a power injector in order to create a virtually blood-free environment (5).

*OCT analysis*

Thin-cap fibroatheroma (TCFA) was defined as a lipid-rich plaque with the thinnest fibrous cap thickness (FCT) <65 µm. FCT was measured 3 times at the thinnest part, and the average value was calculated.

Microchannel was defined as a small black hole with a diameter of 50-100 μm that was present on at least three consecutive cross-sectional frames (6).

Cholesterol crystal was defined as thin linear region of high light intensity, without signal attenuation (7).

The reference area was defined as the mean of the most normal appearing segments 5 mm proximal and distal to the lesion shoulders by OCT. Percentage of area stenosis (AS) was calculated as: (1 – minimal lumen area (MLA) / mean of proximal and distal reference area) x 100%.

In order to identify the presence of local inflammation at the level of culprit plaques, the presence of macrophages infiltrates at the culprit site and along the entire OCT pullback was assessed with both qualitative and quantitative method, as previously describe (8). According to International Working Group for Intravascular Optical Coherence Tomography (IWG-IVOCT) Consensus standards (9), macrophages have been visualized by OCT imaging as signal-rich, distinct, or confluent punctate regions that exceed the intensity of background speckle noise and generate a backward shadowing.

TCFA, microchannel, cholesterol crystal, and macrophage infiltration were recorded only for their presence.

***Additional References***

1. Knuuti J, Wijns W, Saraste A, Capodanno D, Barbato E, Funck-Brentano C, Prescott E, Storey RF, Deaton C, Cuisset T, Agewall S, Dickstein K, Edvardsen T, Escaned J, Gersh BJ, Svitil P, Gilard M, Hasdai D, Hatala R, Mahfoud F, Masip J, Muneretto C, Valgimigli M, Achenbach S, Bax JJ; ESC Scientific Document Group. 2019 ESC Guidelines for the diagnosis and management of chronic coronary syndromes. Eur Heart J. 2020;41:407-477.
2. Thygesen K, Alpert JS, Jaffe AS, Chaitman BR, Bax JJ, Morrow DA, White HD; Executive Group on behalf of the Joint European Society of Cardiology (ESC)/American College of Cardiology (ACC)/American Heart Association (AHA)/World Heart Federation (WHF) Task Force for the Universal Definition of Myocardial Infarction. Fourth Universal Definition of Myocardial Infarction (2018). Circulation. 2018;138:e618-e651.
3. Collet JP, Thiele H, Barbato E, Barthélémy O, Bauersachs J, Bhatt DL, Dendale P, Dorobantu M, Edvardsen T, Folliguet T, Gale CP, Gilard M, Jobs A, Jüni P, Lambrinou E, Lewis BS, Mehilli J, Meliga E, Merkely B, Mueller C, Roffi M, Rutten FH, Sibbing D, Siontis GCM; ESC Scientific Document Group. 2020 ESC Guidelines for the management of acute coronary syndromes in patients presenting without persistent ST-segment elevation. Eur Heart J. 2021;42:1289-1367.
4. Ibanez B, James S, Agewall S, Antunes MJ, Bucciarelli-Ducci C, Bueno H, Caforio ALP, Crea F, Goudevenos JA, Halvorsen S, Hindricks G, Kastrati A, Lenzen MJ, Prescott E, Roffi M, Valgimigli M, Varenhorst C, Vranckx P, Widimský P; ESC Scientific Document Group. 2017 ESC Guidelines for the management of acute myocardial infarction in patients presenting with ST-segment elevation: The Task Force for the management of acute myocardial infarction in patients presenting with ST-segment elevation of the European Society of Cardiology (ESC). Eur Heart J. 2018 Jan 7;39(2):119-177.
5. Tearney GJ, Regar E, Akasaka T, Adriaenssens T, Barlis P, Bezerra HG, Bouma B, Bruining N, Cho JM, Chowdhary S, Costa MA, de Silva R, Dijkstra J, Di Mario C, Dudek D, Falk E, Feldman MD, Fitzgerald P, Garcia-Garcia HM, Gonzalo N, Granada JF, Guagliumi G, Holm NR, Honda Y, Ikeno F, Kawasaki M, Kochman J, Koltowski L, Kubo T, Kume T, Kyono H, Lam CC, Lamouche G, Lee DP, Leon MB, Maehara A, Manfrini O, Mintz GS, Mizuno K, Morel MA, Nadkarni S, Okura H, Otake H, Pietrasik A, Prati F, Räber L, Radu MD, Rieber J, Riga M, Rollins A, Rosenberg M, Sirbu V, Serruys PW, Shimada K, Shinke T, Shite J, Siegel E, Sonoda S, Suter M, Takarada S, Tanaka A, Terashima M, Thim T, Uemura S, Ughi GJ, van Beusekom HM, van der Steen AF, van Es GA, van Soest G, Virmani R, Waxman S, Weissman NJ, Weisz G; International Working Group for Intravascular Optical Coherence Tomography (IWG-IVOCT). Consensus standards for acquisition, measurement, and reporting of intravascular optical coherence tomography studies: a report from the International Working Group for Intravascular Optical Coherence Tomography Standardization and Validation. J Am Coll Cardiol. 2012;59:1058-1072.
6. Uemura S, Ishigami K, Soeda T, Okayama S, Sung JH, Nakagawa H, Somekawa S, Takeda Y, Kawata H, Horii M, Saito Y. Thin-cap fibroatheroma and microchannel findings in optical coherence tomography correlate with subsequent progression of coronary atheromatous plaques. Eur Heart J. 2012;33:78-85.
7. Liu L, Gardecki JA, Nadkarni SK, Toussaint JD, Yagi Y, Bouma BE, Tearney GJ. Imaging the subcellular structure of human coronary atherosclerosis using micro-optical coherence tomography. Nat Med. 2011;17:1010-1014.
8. Ong DS, Lee JS, Soeda T, Higuma T, Minami Y, Wang Z, Lee H, Yokoyama H, Yokota T, Okumura K, Jang IK. Coronary Calcification and Plaque Vulnerability: An Optical Coherence Tomographic Study. Circ Cardiovasc Imaging. 2016;9:e003929.
9. Prati F, Regar E, Mintz GS, Arbustini E, Di Mario C, Jang IK, Akasaka T, Costa M, Guagliumi G, Grube E, Ozaki Y, Pinto F, Serruys PW; Expert's OCT Review Document. Expert review document on methodology, terminology, and clinical applications of optical coherence tomography: physical principles, methodology of image acquisition, and clinical application for assessment of coronary arteries and atherosclerosis. Eur Heart J. 2010;31:401-15.

***Additional Tables***

**Table S1.** Predictors of DMC in the overall population by univariate and multivariate logistic regression analysis.

|  | **Univariate analysis** | | **Multivariable analysis** | |
| --- | --- | --- | --- | --- |
|  | **OR (95% C.I.)** | **p** | **OR (95% C.I.)** | **p** |
| Dyslipidaemia | 0.584 (0.373; 0.916) | **0.019** | 0.661 (0.405; 1.080) | 0.009 |
| Obesity | 0.472 (0.281; 0.795) | **0.005** | 0.543 (0.310; 0.953) | **0.033** |
| Familiar history of CAD | 0.611 (0.372; 1.003) | 0.051 |  |  |
| Diabetes duration (months from T2DM diagnosis) | 1.005 (1.001; 1.009) | **0.019** | 1.004 (0.999; 1.008) | 0.090 |
| Fasting glycaemia | 1.004 (0.999; 1.008) | 0.085 |  |  |
| HbA1c level | 1.031 (1.007; 1.055) | **0.011** | 1.023 (0.988; 1.049) | 0.070 |
| LDL Cholesterol | 0.989 (0.980; 0.998) | **0.014** | 0.988 (0.979; 0.998) | **0.015** |
| Insulin therapy | 3.595 (2.212; 5.845) | **<0.001** | 2.689 (1.547; 4.672) | **<0.001** |
| Metformin therapy | 0.449 (0.283; 0.715) | **0.001** | 0.703 (0.413; 1.199) | 0.196 |

**Legend**: DMC: Diabetic Microvascular Complications; CAD: coronary artery disease; T2DM: Type 2 Diabetes Mellitus; HbA1c: Glycated Haemoglobin; LDL: Low Density Lipoprotein; OR: Odds Ratio; C.I.: Confidence Interval.

**Table S2.** Clinical, echocardiographic and angiographic features in the overall OCT population according to the presence or absence of DMC.

| **Characteristics** | | **Overall OCT population**  (n= 96) | | **OCT population with diabetic microvascular complications**  (n= 46) | | **OCT population without diabetic microvascular complications**  (n = 50) | | **p value** |  |
| --- | --- | --- | --- | --- | --- | --- | --- | --- | --- |
| ***Clinical characteristics*** | |  | |  | |  | |  |  |
| Age [median (IQR)] | | 69.0 [62.0; 76.0] | | 69.5 [63.0; 77.0] | | 66.5 [61.0; 74.2] | | 0.233 |  |
| Male sex [n, (%)] | | 72 (75.0) | | 37 (80.4) | | 35 (70.0) | | 0.238 |  |
| Hypertension [n, (%)] | | 80 (83.3) | | 39 (84.7) | | 41 (82.0) | | 0.715 |  |
| Smoking habit [n, (%)] | | 47 (49.0) | | 24 (52.2) | | 23 (46.0) | | 0.545 |  |
| Dyslipidaemia [n, (%)] | | 55 (57.3) | | 21 (45.6) | | 34 (68.0) | | **0.027** |  |
| Obesity (BMI > 30 kg/m^2^) [n, (%)] | | 24 (25.0) | | 7 (15.2) | | 17 (34.0) | | **0.034** |  |
| Familiar history of CAD [n, (%)] | | 26 (27.1) | | 10 (21.7) | | 16 (32.0) | | 0.258 |  |
| CKD (eGFR < 60 ml/min per 1.73 m^2^) [n, (%)] | | 16 (16.6) | | 7 (15.2) | | 9 (18.0) | | 0.535 |  |
| Clinical presentation [n, (%)] | |  | |  | |  | | 0.344 |  |
| ACS [n, (%)] | | 35 (36.5) | | 19 (41.3) | | 16 (32.0) | |  |  |
| CCS [n, (%)] | | 61 (63.5) | | 27 (58.7) | | 34 (68.0) | |  |  |
| Diabetes duration (months since T2DM diagnosis) [median (IQR)] | | 84 [60; 96] | | 90 [72; 108] | | 72 [60; 96] | | **0.025** |  |
| ***Diabetic complications*** | |  | |  | |  | |  |  |
| Carotid arterial disease [n, (%)] | | 49 (51.0) | | 21 (45.6) | | 28 (56.0) | | 0.311 |  |
| Peripheral arterial disease [n, (%)] | | 16 (16.7) | | 11 (23.9) | | 5 (10.0) | | 0.068 |  |
| Previous stroke/TIA [n, (%)] | | 6 (6.2) | | 2 (4.3) | | 4 (8.0) | | 0.460 |  |
| Diabetic retinopathy [n, (%)] | | 32 (33.3) | | 32 (69.6) | | - | | - |  |
| Diabetic neuropathy [n, (%)] | | 27 (28.1) | | 27 (58.7) | | - | | - |  |
| Diabetic nephropathy [n, (%)] | | 35 (36.5) | | 35 (76.1) | | - | | - |  |
| ***Laboratory data*** | |  | |  | |  | |  |  |
| Fasting glycaemia (mg/dL) [median (IQR)] | | 132.5 [111; 160] | | 135.5 [116; 171.5] | | 126.5 [108.2; 150.7] | | 0.429 |  |
| HbA1c (mmol/mol) [median (IQR)] | | 48 [43; 53] | | 50.5 [47.0; 58.5] | | 45.0 [40.7; 51.0] | | **0.019** |  |
| Total Cholesterol (mg/dL) [median (IQR)] | | 154.0 [126.5; 178] | | 150.5 [127.5; 178.0] | | 157.5 [125.7; 178.2] | | 0.931 |  |
| LDL Cholesterol (mg/dL) [median (IQR)] | | 74.5 [61.0; 90.7] | | 67.5 [57.7; 80.5] | | 79.5 [66.5; 94.0] | | 0.092 |  |
| Hb (g/dL) [median (IQR)] | | 12.7 [11.7; 13.7] | | 12.9 [11.8; 13.6] | | 12.5 [11.7; 13.9] | | 0.536 |  |
| WBC (x10^3^/L) [median (IQR)] | | 7.7 [7.1; 9.1] | | 7.7 [6.8; 9.0] | | 7.7 [7.1; 9.1] | | 0.636 |  |
| PLT (x10^3^/L) [median (IQR)] | | 240.0 [199.2; 266.0] | | 240.0 [202.5; 266.7] | | 240.0 [189.0; 268.5] | | 0.913 |  |
| Serum creatinine on admission (mg/dL) [median (IQR)] | | 0.92 [0.80; 1.08] | | 0.97 [0.86; 1.14] | | 0.86 [0.78; 0.96] | | **0.014** |  |
| Troponin T peak (ng/mL) [median (IQR)] | | 0.06 [0.01; 0.39] | | 0.06 [0.01; 0.51] | | 0.06 [0.01; 0.32] | | 0.479 |  |
| ***Echocardiographic data*** | |  | |  | |  | |  |  |
| LVEF on admission (%) [median (IQR)] | | 58.5 [52.0; 61.0] | | 58.0 [52.0; 61.0] | | 59.0 [51.7; 61.0] | | 0.696 |  |
| LVEF on admission < 50% [n, (%)] | | 17 (17.7) | | 8 (17.4) | | 9 (18.0) | | 0.938 |  |
| Diastolic dysfunction [n, (%)] | | 76 (79.2) | | 41 (89.1) | | 35 (70.0) | | **0.021** |  |
| Grade II or III diastolic dysfunction [n, (%)] | | 21 (21.9) | | 13 (28.2) | | 6 (16.0) | | **0.009** |  |
| E/e’ [median (IQR)] | | 8 [7; 9] | | 8 [7; 12] | | 8 [7; 8] | | **0.027** |  |
| ***Therapy at admission*** | |  | |  | |  | |  |  |
| Insulin [n, (%)] | | 31 (32.3) | | 22 (47.8) | | 9 (18.0) | | **0.002** |  |
| Metformin [n, (%)] | | 65 (67.7) | | 29 (63.9) | | 36 (72.0) | | 0.348 |  |
| Sulfonylureas [n, (%)] | | 30 (31.2) | | 13 (28.3) | | 17 (34.0) | | 0.544 | |
| GLP1/DPP4-I [n, (%)] | | 15 (15.6) | | 7 (15.2) | | 8 (16.0) | | 0.916 | |
| SGLT-2-Inhibitors [n, (%)] | | - | | - | | - | | - | |
| Aspirin [n, (%)] | | 59 (61.5) | | 32 (69.6) | | 27 (54.0) | | 0.117 | |
| Beta-blockers [n, (%)] | | 36 (37.5) | | 19 (41.3) | | 17 (34.0) | | 0.460 |  |
| CCBs [n, (%)] | | 18 (18.8) | | 9 (19.6) | | 9 (18.0) | | 0.844 |  |
| ACE-i/ARBs [n, (%)] | | 54 (56.2) | | 26 (56.5) | | 28 (56.0) | | 0.959 |  |
| Statin [n, (%)] | | 53 (55.2) | | 28 (60.9) | | 25 (50.0) | | 0.285 |  |
| Diuretics [n, (%)] | | 26 (29.3) | | 13 (28.3) | | 13 (26.0) | | 0.803 |  |
| ***Therapy at dismission*** | |  | |  | |  | |  |  |
| Insulin [n, (%)] | | 39 (40.6) | | 27 (58.7) | | 12 (24.0) | | **<0.001** |  |
| Metformin [n, (%)] | | 70 (72.9) | | 31 (67.4) | | 39 (78.0) | | 0.242 |  |
| Sulfonylureas [n, (%)] | | 28 (29.1) | | 12 (26.1) | | 16 (32.0) | | 0.524 | |
| GLP1/DPP4-I [n, (%)] | | 13 (13.5) | | 6 (13.0) | | 7 (14.0) | | 0.891 | |
| SGLT-2-Inhibitors [n, (%)] | | - | | - | | - | | - | |
| Aspirin [n, (%)] | | 90 (93.7) | | 42 (91.3) | | 48 (96.0) | | 0.342 | |
| Beta-blockers [n, (%)] | | 88 (91.7) | | 41 (89.1) | | 47 (94.0) | | 0.388 |  |
| CCBs [n, (%)] | | 20 (20.8) | | 11 (23.9) | | 9 (18.0) | | 0.476 |  |
| ACE-i/ARBs [n, (%)] | | 87 (90.6) | | 41 (89.1) | | 46 (92.0) | | 0.629 |  |
| Statin [n, (%)] | | 91 (94.79) | | 44 (45.8) | | 47 (94.0) | | 0.715 |  |
| Diuretics [n, (%)] | | 27 (28.1) | | 13 (28.3) | | 14 (28.0) | | 0.977 |  |
| ***Angiographic data*** | |  | |  | |  | |  |  |
| Multivessel CAD [n, (%)] | | 48 (50.0) | | 28 (60.9) | | 20 (40.0) | | **0.041** |  |

**Legend to table:**

OCT: Optical Coherence Tomography; DMC: Diabetic Microvascular Complications; IQR: InterQuartile Range; BMI: body mass index; CAD: Coronary Artery Disease; CKD: Chronic Kidney Disease; GFR: Glomerular Filtration Rate; ACS: Acute Coronary Syndromes; CCS: Chronic Coronary Sindromes; T2DM: Type 2 Diabetes Mellitus; TIA: Transient Ischemic Attack; HbA1c: Glycated Haemoglobin; LDL: Low-Density Lipoprotein; Hb: Haemoglobin; WBC: White Blood Count; PLT: Platelets; LVEF: Left Ventricle Ejection Fraction; GLP-1: Glucagon-Like Peptide-1; DPP4-I: DiPeptidyl Peptidase-4 Inhibitors; SGLT-2: Sodium-Glucose co-transpoter-2; CCBs: Calcium-Channels Blockers; ACEi: angiotensin converting enzymes inhibitors; ARBs: Angiotensin receptor blockers.

**Table S3.** Sensitivity analysis of OCT characteristics of coronary plaques in the culprit vessel according to the presence or absence of diabetic microvascular complications excluding patients with diabetic neuropathy.

| **Characteristics** | | **Plaques of patients with diabetic microvascular complications**  (n= 43) | **Plaques of patients without diabetic microvascular complications**  (n = 85) | **p value** |
| --- | --- | --- | --- | --- |
| ***Clinical characteristics*** |  | |  |  |
| Plaque vessel |  | |  | 0.212 |
| LAD [n, (%)] | 30 (69.8) | | 71 (83.6) |  |
| LCx [n, (%)] | 10 (23.3) | | 7 (8.2) |  |
| RCA [n, (%)] | 3 (7.0) | | 7 (8.2) |  |
| Plaque type |  | |  | **0.029** |
| Fibrous plaque [n, (%)] | 24 (55.8) | | 27 (31.8) |  |
| Lipid plaque [n, (%)] | 19 (44.2) | | 58 (68.2) |  |
| FCT (µm) [median (IQR)] | 100.0 (75.0 – 115.0) | | 100.0 [82.5; 110.0] | 0.797 |
| Lipid arc mean (°) [median (IQR)] | 84.9 [53.4; 167.7] | | 146.5 [73.6; 203.0] | **0.045** |
| Lipid length (mm) (mean ± standard deviation) | 6.6 ± 3.6 | | 7.5 ± 4.0 | 0.217 |
| Lipid index (mm) (mean ± standard deviation) | 834.3 ± 761.3 | | 1196.2 ± 961.6 | 0.057 |
| TCFA [n, (%)] | 7 (16.3) | | 13 (15.3) | 0.879 |
| Calcifications [n, (%)] | 29 (67.4) | | 35 (41.2) | **0.031** |
| Calcific arc mean (°) (mean ± standard deviation) | 135.9 ± 70.2 | | 110.5 ± 108.4 | 0.327 |
| Calcific length (mm) (mean ± standard deviation) | 9.8 ± 5.5 | | 7.9 ± 4.1 | 0.062 |
| Calcific depth (mm) (mean ± standard deviation) | 0.68 ± 0.27 | | 0.52 ± 0.24 | **0.010** |
| Spotty calcium [n, (%)] | 12 (27.9) | | 37 (43.5) | 0.184 |
| Plaque length (mm) (mean ± standard deviation) | 14.0 ± 6.9 | | 13.5 ± 5.9 | 0.621 |
| Healed plaques [n, (%)] | 10 (23.3) | | 7 (8.2) | **0.014** |
| Macrophages [n, (%)] | 15 (34.9) | | 35 (41.2) | 0.527 |
| Microvessels [n, (%)] | 17 (39.5) | | 29 (34.1) | 0.570 |
| Cholesterol crystals [n, (%)] | 13 (30.2) | | 21 (24.7) | 0.547 |
| MLA (mm^2^) (mean ± standard deviation) | 3.8 ± 2.2 | | 3.9 ± 2.1 | 0.752 |
| AS (mm^2^) (mean ± standard deviation) | 59.3 ± 11.7 | | 58.8 ± 12.1 | 0.792 |

**Legend to table:**

IQR: InterQuartile Range; LAD: left anterior descending artery; LCx: left circumflex; RCA: right coronary artery; OCT: optical coherence tomography; FCT: fibrous cap thickness; TCFA: thin cap fibroatheroma; MLA; minimal lumen area; AS: area stenosis.

**Table S4.** Predictors of large calcifications in the OCT sub-group by univariate and multivariate logistic regression analysis.

|  | **Univariate analysis** | | **Multivariate analysis** | |
| --- | --- | --- | --- | --- |
|  | **OR (95% C.I.)** | **p** | **OR (95% C.I.)** | **p** |
| Male sex | 3.714 (1.404; 9.828) | **0.008** | 3.716 (1.091; 12.655) | **0.036** |
| Familiar History of CAD | 0.413 (0.173; 0.988) | **0.047** | 0.328 (0.114; 0.945) | **0.039** |
| Creatinine, per unit | 7.395 (1.185; 46.137) | **0.032** | - | ns |
| Multivessel disease | 2.878 (1.281; 6.486) | **0.011** | - | ns |
| Diabetes duration (months from T2DM diagnosis) | 1.012 (0.995; 1.030) | 0.177 | - | ns |
| Presence of at least one of DMC | 4.887 (2.098; 11.385) | **<0.001** | 3.838 (1.505; 9.786) | **0.005** |
| Clinical presentation (ACS) | 1.057 (0.477; 2.346) | 0.891 | - | ns |

**Legend to Table**: ACS. Acute Coronary Syndrome. OCT. Optical Coherence Tomography; OR: Odds Ratio; C.I.: Confidence Interval; CAD: Coronary Artery Disease; T2DM: Type 2 Diabetes Mellitus; DMC: Diabetic Microvascular Complications; ns: non-significant.

All variables in Table 2 and the presence of at least one of diabetic microvascular complications have been tested to predict calcifications, although only variables with p-value <0.050, diabetes duration (months from T2DM diagnosis) and clinical presentation have been shown in the table. Variables that were significantly related to coronary calcification, months from T2DM diagnosis and clinical presentation have been included in multivariate analysis.

**Table S5.** Predictors of healed plaque phenotype in the OCT sub-group by univariate and multivariate logistic regression analysis.

|  | **Univariate analysis** | | **Multivariate analysis** | |
| --- | --- | --- | --- | --- |
|  | **OR (95% C.I.)** | **p** | **OR (95% C.I.)** | **p** |
| Total cholesterol, per unit | 1.010 (1.001; 1.019) | **0.026** | - | ns |
| Diabetes duration (months from T2DM diagnosis) | 1.011 (0.994; 1.029) | 0.207 | - | ns |
| Presence of at least one of DMC | 3.257 (1.350; 7.859) | **0.009** | 3.213 (1.299; 7.949) | **0.012** |
| Clinical presentation (ACS) | 0.671 (0.291; 1.544) | 0.348 | - | ns |

**Legend to Table**: ACS. Acute Coronary Syndrome. OCT: Optical Coherence Tomography; OR: Odds Ratio; C.I.: Confidence Interval; T2DM: Type 2 Diabetes Mellitus; DMC: Diabetic Microvascular Complications; ns: non-significant.

All variables in Table 2 and the presence of at least one of diabetic microvascular complications have been tested to predict layered phenotype, although only variables with p-value < 0.050, diabetes duration (months from T2DM diagnosis) and clinical presentation have been shown in the table. Variables that were significantly related to healed plaque phenotype, months from T2DM diagnosis and clinical presentation have been included in multivariate analysis.

**Table S6.** Predictors of lipid plaque phenotype in the OCT sub-group by univariate and multivariate logistic regression analysis.

|  | **Univariate analysis** | | **Multivariate analysis** | |
| --- | --- | --- | --- | --- |
|  | **OR (95% C.I.)** | **p** | **OR (95% C.I.)** | **p** |
| Insulin therapy on admission | 0.451 (0.209; 0.973) | **0.042** | - | ns |
| Statin therapy on admission | 0.360 (0.130; 0.997) | **0.049** | - | ns |
| Diabetes duration (months from T2DM diagnosis) | 0.980 (0.964; 0.997) | **0.021** | - | ns |
| Presence of at least one of DMC | 0.272 (0.126; 0.586) | **0.001** | 0.383 (0.160; 0.918) | **0.031** |
| Clinical presentation (ACS) | 0.977 (0.461; 2.071) | 0.952 | - | ns |

**Legend to Table**: ACS. Acute Coronary Syndrome. OCT: Optical Coherence Tomography; OR: Odds Ratio; C.I.: Confidence Interval; T2DM: Type 2 Diabetes Mellitus; DMC: Diabetic Microvascular Complications; ns: non-significant

All variables in Table 2 and the presence of at least one of diabetic microvascular complications have been tested to predict layered phenotype, although only variables with p-value < 0.050, diabetes duration (months from T2DM diagnosis) and clinical presentation have been shown in the table. Variables that were significantly related to lipid plaque phenotype, months from T2DM diagnosis and clinical presentation have been included in multivariate analysis.

**Table S7.** Sensitivity analysis of clinical outcome according to the presence or absence of diabetic microvascular complications including only patients on therapy with metformin at the time of discharge.

| **Characteristics** | **Patients on therapy with metformin at the time of discharge** (n=208) | **Presence of**  **DMC**  (n=98) | **Absence of**  **DMC**  (n=110) | **p value** |
| --- | --- | --- | --- | --- |
| MACEs [n, (%)] | 25 (12.0) | 15 (15.3) | 10 (9.1) | **0.025** |
| CV Death [n, (%)] | 4 (1.9) | 3 (3.1) | 1 (0.9) | 0.133 |
| Non-fatal MI [n, (%)] | 10 (4.8) | 5 (5.1) | 5 (4.5) | 0.489 |
| Planned coronary revascularization [n, (%)] | 11 (5.3) | 7 (7.1) | 4 (3.6) | 0.069 |
| Follow-up time [mean ± standard deviation] | 33.8 ± 15.8 | 31.4 ± 15.6 | 35.9 ± 16.1 | **0.043** |
|  |  |  |  |  |

**Legend to table:** DMC: Diabetic Microvascular Complications; MACEs; Major Adverse Cardiovascular Events; CV: Cardiovascular; MI: Myocardial Infarction.

**Table S8.** Sensitivity analysis of predictors of MACEs including only patients on therapy with metformin at the time of discharge by univariate and multivariate Cox regression analysis.

|  | **Univariate analysis** | | **Multivariable analysis** | |
| --- | --- | --- | --- | --- |
|  | **HR (95% C.I.)** | **p** | **HR (95% C.I.)** | **p** |
| Presence of ≥ 1 DMC | 2.442 (1.089; 5.476) | **0.030** | 2.752 (1.146; 6.609) | **0.024** |
| Male sex | 1.343 (0.536; 3.365) | 0.529 | 1.571 (0.614; 4.015) | 0.346 |
| Age | 0.987 (0.943; 1.033) | 0.564 | 0.987 (0.942; 1.034) | 0.580 |
| LVEF on admission | 1.014 (0.965; 1.065) | 0.579 | 1.014 (0.966; 1.063) | 0.577 |
| Multivessel CAD | 1.191 (0.542; 2.620) | 0.664 | 0.973 (0.427; 2.218) | 0.949 |
| Diabetes duration (months since T2DM diagnosis) | 0.966 (0.992; 1.009) | 1.000 | 0.997 (0.989; 1.006) | 0.552 |

**Legend**: MACEs: Major Adverse Cardiovascular Events; DMC: Diabetic Microvascular Complications; LVEF: Left Ventricle Ejection Fraction; CAD: Coronary Artery Disease; HR: Hazard Ratio; C.I.: Confidence Interval.

All variables in Table 1 and the presence of at least one of DMC have been tested to predict MACEs, although only variables with p-value <0.050, age, male sex, EF on admission, multivessel CAD and diabetes duration (months since T2DM diagnosis) have been shown in the table. Variables that were significantly related to MACEs, age, male sex, EF on admission, multivessel CAD and diabetes duration (months since T2DM diagnosis) have been included in multivariate analysis.

**Table S9.** Sensitivity analysis of clinical outcome according to the presence or absence of diabetic microvascular complications including only patients with ACS as clinical presentation.

| **Characteristics** | **Patients with ACS as clinical presentation** (n=128) | **Presence of**  **DMC**  (n=68) | **Absence of**  **DMC**  (n=60) | **p value** |
| --- | --- | --- | --- | --- |
| MACEs [n, (%)] | 16 (12.5) | 11 (16.2) | 5 (8.3) | **0.010** |
| CV Death [n, (%)] | 3 (2.3) | 2 (2.9) | 1 (1.7) | 0.304 |
| Non-fatal MI [n, (%)] | 4 (3.1) | 3 (4.4) | 1 (1.7) | 0.171 |
| Planned coronary revascularization [n, (%)] | 9 (7.0) | 6 (8.8) | 3 (5.0) | 0.054 |
| Follow-up time [mean ± standard deviation] | 32.2 ± 15.7 | 30.3 ± 15.6 | 34.4 ± 15.6 | 0.158 |
|  |  |  |  |  |

**Legend to table:** ACS: Acute Coronary Syndrome; DMC: Diabetic Microvascular Complications; MACEs; Major Adverse Cardiovascular Events; CV: Cardiovascular; MI: Myocardial Infarction.

**Table S10.** Sensitivity analysis of predictors of MACEs in the ACS population by univariate and multivariate Cox regression analysis.

|  | **Univariate analysis** | | **Multivariable analysis** | |
| --- | --- | --- | --- | --- |
|  | **HR (95% C.I.)** | **p** | **HR (95% C.I.)** | **p** |
| Presence of ≥ 1 DMC | 3.756 (1.280; 11.021) | **0.016** | 3.586 (1.146; 11.218) | **0.028** |
| Smoking habit | 5.922 (1.843; 19.028) | **0.003** | 5.659 (1.419; 22.575) | **0.014** |
| Male sex | 1.213 (0.391; 3.769) | 0.738 | 0.802 (0.218; 2.953) | 0.740 |
| Age | 0.958 (0.905; 1.014) | 0.142 | 0.991 (0.929; 1.057) | 0.784 |
| LVEF on admission | 1.018 (0.960; 1.080) | 0.555 | 1.022 (0.964; 1.084) | 0.459 |
| Multivessel CAD | 0.963 (0.358; 2.592) | 0.941 | 0.982 (0.304; 3.174) | 0.976 |
| Diabetes duration (months since T2DM diagnosis) | 0.992 (0.979; 1.005) | 0.246 | 0.997 (0.984; 1.010) | 0.652 |

**Legend**: ACS: Acute Coronary Syndrome; MACEs: Major Adverse Cardiovascular Events; DMC: Diabetic Microvascular Complications; LVEF: Left Ventricle Ejection Fraction; CAD: Coronary Artery Disease; HR: Hazard Ratio; C.I.: Confidence Interval.

All variables in Table 1 and the presence of at least one of DMC have been tested to predict MACEs, although only variables with p-value <0.050, age, male sex, EF on admission, multivessel CAD and diabetes duration (months since T2DM diagnosis) have been shown in the table. Variables that were significantly related to MACEs, age, male sex, EF on admission, multivessel CAD and diabetes duration (months since T2DM diagnosis) have been included in multivariate analysis.

**Table S11.** Sensitivity analysis of clinical outcome according to the presence or absence of diabetic microvascular complications including only patients with CCS as clinical presentation.

| **Characteristics** | **Patients with CCS as clinical presentation** (n=192) | **Presence of**  **DMC**  (n=104) | **Absence of**  **DMC**  (n=88) | **p value** |
| --- | --- | --- | --- | --- |
| MACEs [n, (%)] | 21 (10.9) | 14 (13.5) | 7 (8.0) | 0.084 |
| CV Death [n, (%)] | 1 (0.5) | 1 (1.0) | 0 (0.0) | 0.317 |
| Non-fatal MI [n, (%)] | 8 (4.2) | 3 (2.9) | 5 (5.7) | 0.615 |
| Planned coronary revascularization [n, (%)] | 12 (6.3) | 7 (7.1) | 4 (3.6) | **0.016** |
| Follow-up time [mean ± standard deviation] | 34.3 ± 15.5 | 33.2 ± 14.4 | 35.5 ± 16.6 | 0.279 |
|  |  |  |  |  |

**Legend to table:** CCS: Chronic Coronary Syndrome; DMC: Diabetic Microvascular Complications; MACEs; Major Adverse Cardiovascular Events; CV: Cardiovascular; MI: Myocardial Infarction.

**Table S12.** Sensitivity analysis of predictors of MACEs in the CCS population by univariate and multivariate Cox regression analysis.

|  | **Univariate analysis** | | **Multivariable analysis** | |
| --- | --- | --- | --- | --- |
|  | **HR (95% C.I.)** | **p** | **HR (95% C.I.)** | **p** |
| Presence of ≥ 1 DMC | 2.187 (0.878; 5.448) | 0.093 | - | **-** |
| Male sex | 0.879 (0.340; 2.268) | 0.789 | - | **-** |
| Age | 0.976 (0.927; 1.026) | 0.340 | - | **-** |
| LVEF on admission | 1.024 (0.964; 1.088) | 0.440 | - | **-** |
| Multivessel CAD | 0.934 (0.396; 2.202) | 0.875 | - | **-** |
| Diabetes duration (months since T2DM diagnosis) | 0.946 (0.993; 1.006) | 1.000 | - | **-** |

**Legend**: CCS: Chronic Coronary Syndrome; MACEs: Major Adverse Cardiovascular Events; DMC: Diabetic Microvascular Complications; LVEF: Left Ventricle Ejection Fraction; CAD: Coronary Artery Disease; HR: Hazard Ratio; C.I.: Confidence Interval.

All variables in Table 1 and the presence of at least one of DMC have been tested to predict MACEs, although only variables with p-value <0.050, age, male sex, EF on admission, multivessel CAD and diabetes duration (months since T2DM diagnosis) have been shown in the table.

***Additional Figures***

**Figure S1**


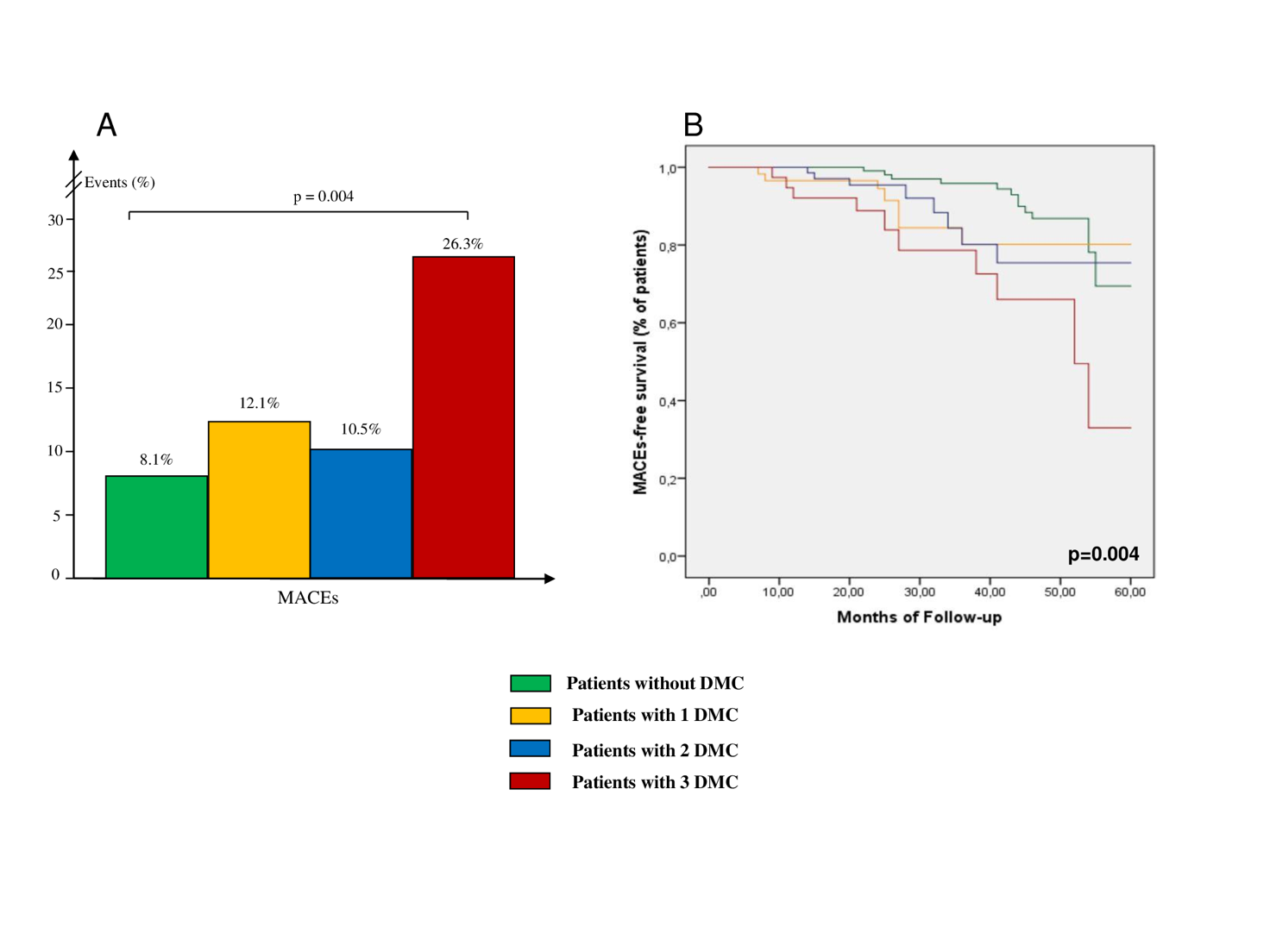


***Additional Figure Legend***

**Figure S1: Panel A:** Clinical outcomes in the overall population stratified according to the number of DMC. **Panel B:** Kaplan–Meier curve for MACEs according to the number of DMC. *Abbreviations:* MACEs; Major Adverse Cardiovascular Events; DMC: Diabetic Microvascular Complications.
